# Gender differences and climate zones in overweight and obesity prevalence in European elementary school children from 2000 to 2020: a systematic review and meta-analysis

**DOI:** 10.3389/fpubh.2023.1198877

**Published:** 2023-09-21

**Authors:** Nikola Prvulović, Miodrag Djordjević, Saša Pantelić

**Affiliations:** ^1^Faculty of Sport and Physical Education, University of Niš, Niš, Serbia; ^2^Faculty of Science and Mathematics, University of Niš, Niš, Serbia

**Keywords:** obesity, prevalence, climate zone, global warming, trend, meta analysis, meta regression

## Abstract

**Introduction:**

After 2000, there are more obese than underweight people in the world. We face a rapid increase in average global warming of 1.5°C, reported as a syndemic problem of three interconnected epidemics: obesity, global warming, and undernutrition. We aimed to analyze the impact and association between global warming and obesity in children and differences by gender across Europe after 2000.

**Methods:**

We searched PubMed, MEDLINE, Google Scholar, ScienceDirect, and Embase databases. The considered population were children aged 6–14. Only cross-sectional studies that defined obesity by the IOTF cutoffs and the subjects’ place of residence, used to determine precise climate zones, were included. We assessed the prevalence of obesity and overweight using a random-effects and the Mantel–Haenszel fixed-effect method when heterogeneity was greater/lower than 50%. We did a subgroup analysis for prevalence across gender, obesity, and overweight, two decades, regions, countries, and the Köppen–Geiger climate zones. Random effects of the meta-regression were used to study the global warming impact and differences in trends across European countries by gender for both conditions separately.

**Results:**

We identified 114 studies that included 985,971 children from 39 European countries. A significant difference between genders was in favor of obese girls 4.78 (95% CI: 3.85–5.93) versus boys 5.76% (95% CI: 5.11–6.48, *p* = 0.03), respectively, but not for overweight children. Most of the obese girls were in South Europe 7.51% (95% CI: 6.61–8.51) versus East Europe 2.86% (95% CI: 23–3.12), versus boys in South Europe 8.66% (95% CI: 7.68–9.74) and North Europe 3.49% (95% CI: 2.90–4.19), respectively. The “cold” Köppen–Geiger climate zone, with lowest temperatures, has the largest trend rise between two decades of 2.8% and 1.53% for obese girls and boys, and 5.31% and 1.81% for overweight girls and boys, respectively, followed by the smallest number of obese girls 3.28% (95% CI: 2.17–4.92) and boys 3.58% (95% CI: 2.39–5.33), versus the zone with the highest temperatures “hot” for girls 7.02% (95% CI: 6.30–7.82) and for boys 8.23% (95% CI: 7.55–8.96), respectively. The meta-regression proved global warming has a significant impact on the distribution of obesity and overweight across climate zones, *R*^2^ = 0.52 and *R*^2^ = 0.22. No significant gender differences, or significant interaction, was noted.

**Conclusion:**

Our meta-analysis provides a comprehensive overview of the association between and impact of global warming on obesity. This impact increases obesity among children in Europe throughout all climate zones, and emphasizes an urgent call for further preventive methods in schools, since obesity differences continue their trend of disappearing into the future.

**Systematic review registration**: https://www.crd.york.ac.uk/prospero/display_record.php?ID=CRD42021282127, identifier: CRD42021282127.

## 1. Introduction

The prevalence of obesity (OB) has tripled since 1975. Alarmingly, following 2000, humanity experienced a watershed moment when the number of overweight (OW) individuals exceeded the number of underweighted individuals (1, 2). In 2015, across 175 countries, there were 107.7 million children with obesity, equivalent to 5% (3). Furthermore, 340 million children aged five to 19 were with OW or with obesity in 2016 (2), and in 2019 there were 398,000 children, or 2.9% with severe obesity aged six to nine in Europe (4). Even though there is a difference in the age cut-off points for children according to the WHO standard for ages five to 19, and the IOTF for ages two to 18, there are differences in the values of BMI among children younger and older than five. A major cause is inclusive obligatory physical activity in elementary school (5). Physical activity is one of the best methods for the prevention of OB, which has the greatest impact and positive effects on the reduction and maintenance of normal weight both in children and adults (1, 6–9). The results also indicate that elementary school children take part in physical education at a rate of 97.8% (10). Systematic review studies and meta-analyses provided the data needed to create better solutions to the problem of OB (11–13). Even though there are numerous meta-analyses, some of them are outdated (14, 15), some focused on only one country (16), and some had an extensive age range for the subjects (13, 17).

Since 2000, the surface area of Europe has become warmer compared to the global average by 1.2°C in the first decade, 1.7°C to 1.9°C in the second decade compared to the pre-industrial level (18). Due to missing information on precise temperature changes across countries, the available data indicate the same temperature increase in all European countries of 1.5°C (18). The most recent reports indicate a syndemic problem of three mutually connected epidemics: obesity, global warming, and undernutrition, which are virtually under-researched (19). There is a bidirectional relationship between adiposity and global warming. With rising atmospheric air temperatures, people typically will have less adaptive thermogenesis and become less physically active (20). Deeper analysis showed the same principle of spending more energy for digesting cold food than hot the owners of a microwave have an increase of 2.1 kg in weight (21), in order to bring it up to the body temperature (22), and spending more energy to increase the temperature of air in the lungs (23). In laboratory experiments subjects expend more energy when the ambient temperature decreases even by a few degrees (24). This meta-analysis studied gender differences in the prevalence of obesity among elementary school children from 39 European countries, two interacting epidemics, and the state of OB among children across the climate zones of Europe that have a positive warming trend after 2000.

## 2. Methods

**Figure 1:**
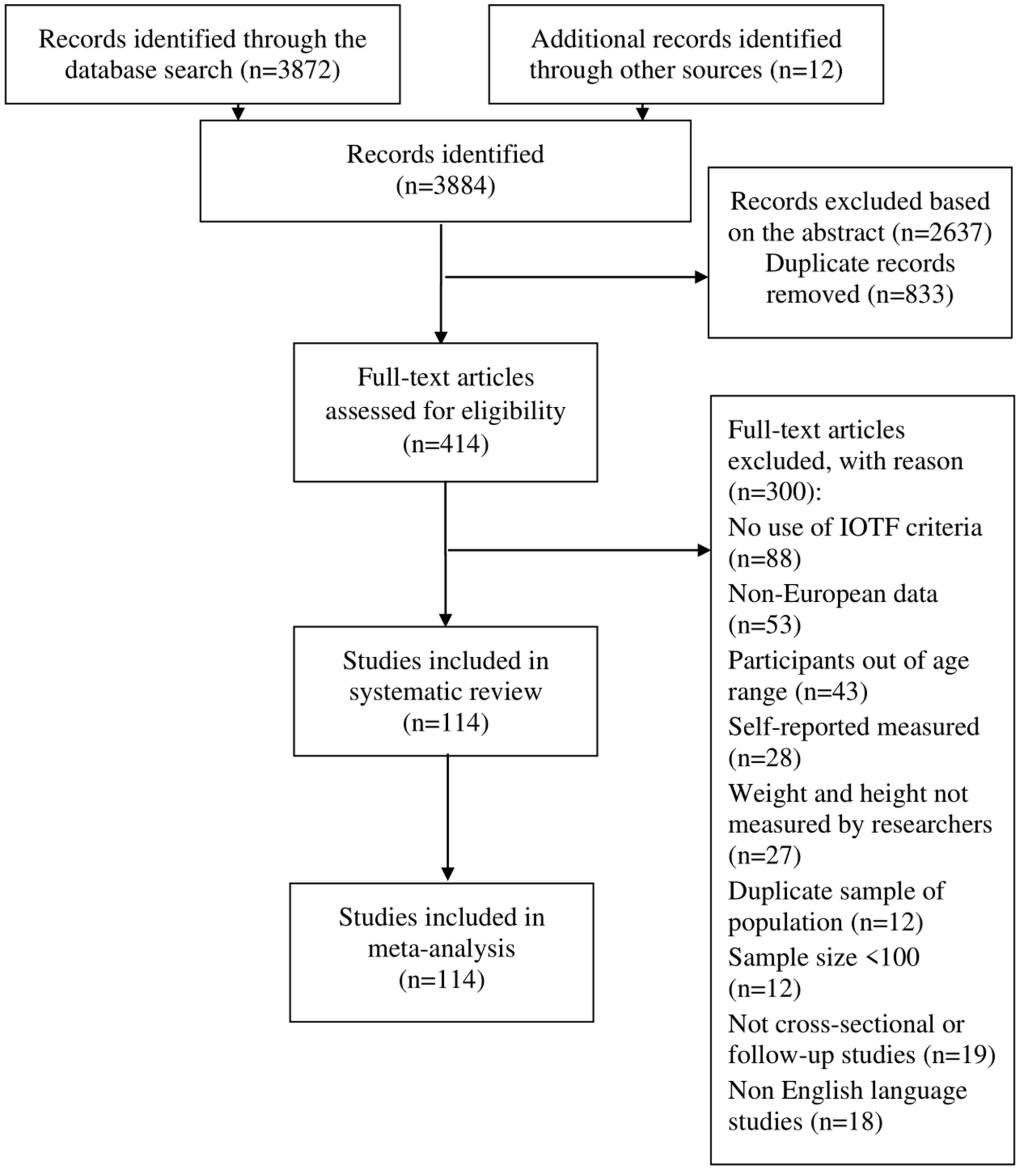
PRISMA flow diagram of the study selection process.

We followed the meta-analysis of observational studies in epidemiology (MOOSE) (25), and Preferred Reporting Items for Systematic Reviews and Meta–analyses (PRISMA) reporting guidelines (26) (Figure 1). The protocol for this review was registered and published on the Prospero database (registration number: CRD42021282127).

### 2.1. Data sources and search strategies

To analyze the existing literature, the following electronic databases were searched for papers published between 2000 and 2020: PubMed, MEDLINE, Google Scholar, ScienceDirect, and Embase. The following terms were combined to design the search strategy: (1) population (children, childhood, schoolchildren, adolescent, school-aged); (2) outcome (obesity, overweight, body composition, weight status); (3) study design and type of study (prevalence, cross-sectional, trend, observational, epidemiology); and (4) location (includes terms for different European countries, *n* = 39) (see Supplementary Table S1). The research strategy was modified for each electronic database, where possible, with the aim of increasing sensitivity. All the titles and abstracts were reviewed for potential studies which could be included in the systematic review and meta-analysis. In addition, the lists of references of previous review and original research papers were also analyzed. Relevant studies were obtained following a detailed review based on inclusion and exclusion criteria.

### 2.2. Inclusion criteria

#### 2.2.1. Type of study

The inclusion criteria were (1) studies reporting a population-based prevalence of OW or OB according to the BMI calculated as weight in kg divided by height in m^2^, and cut-offs proposed by the IOTF (27, 28) (the IOTF referent cut-off points and specific IOTF cut-off points), BMI for OW boys aged from 6 to 14 years is (17.55 to 22.62), and for girls (17.34 to 23.34) (27). And specific IOTF cut-off points, BMI for OW boys aged from 6 to 14 years is (17.52 to 22.60), and for girls (17.33 to 23.27) (28). BMI for OB boys aged from 6 to 14 years is (19.78 to 27.63), and for girls (19.65 to 28.57) (27). And specific IOTF cut-off points, BMI for OB boys aged from 6 to 14 years is (19.76 to 27.64), and for girls (19.62 to 28.42) (28). For detailed results see in (27, 28). Six studies had the same referent cut-off points but with different names; (2) cross-sectional studies with weight and height measured by trained personnel; and (3) studies including populations aged six to 14.

### 2.3. Exclusion criteria

The exclusion criteria were the following: (1) studies published in languages other than English; (2) the sample was less than 250 children (on the rationale that smaller samples may lead to a lack of precision in the prevalence estimates) (17); (3) they were duplicate reports of the same study; and (4) studies that do not have data of permanent residence of the subjects.

### 2.4. Search and data extraction

In order to identify suitable studies that meet the criteria, three authors worked independently, at the end of collecting a given number of collected studies, the three authors checked each other for possible duplicate works and checked the found works, the first author checked the found studies of the second and third author, while both co-authors checked works of the first author. After that, each author will independently extract data from his found works. The main characteristics of the selected studies are summarized in Supplementary Table S2, including information regarding the (1) reference number (name of author and year); (2) survey period; (3) subject characteristics (age and sample size); (4) size by gender; (5) results by gender for prevalence of OW and OB; (6) region (29); (7) country; (8) country latitude;^1^ and (9) climate zones according to Köppen–Geiger (30), (for every study which contained origin data for the sample, the appropriate climate zone was noted).

### 2.5. Quality assessment

The Joanna Briggs Institute tool (31) was used to evaluate the risk of bias in the prevalence studies. This tool consists of a rating list with 10 criteria, which can be assessed as yes (coded as 1), no (coded as 0), not applicable (coded as NA), or unclear (coded as?); thus, the score for each study ranged from 0 to 10. Depending on its score, we rated each study as low risk (7–10), moderate risk (4–6), or high risk of bias (1–3). (See Supplementary Table S3).

### 2.6. Statistical analysis

Data were obtained from cross-sectional studies to estimate pooled prevalence means. The statistical and meta-analysis were carried out using the comprehensive meta-analysis program, version 3. During data entry, to estimate the prevalence rate, the matrix was designed using the estimate rate method with raw data (32). After obtaining results for a general point estimate, the subgroups were further analyzed based on gender. For one analysis they were treated as separate units, and when necessary for a different analysis, as one. Categorical and integrational moderators were used to obtain the data for the prevalence rate per region (*n* = 6) (29), and country (*n* = 39) presented over time, that is, two groups per Time interval (two decades 2000–2009 and 2010–2020), four climate zones according to Köppen–Geiger (hot-Csa, warm-Cfb, temperate-Dfb and cold-Dfc), [for a detailed explanation see (30)], and latitude. Some countries have two or three different climate zones, such as Romania, Switzerland, Sweden, Bulgaria, Montenegro, etc. (30), so when determining the climate zone, precise latitude data were used for the cities and regions of origin of the subjects, if provided (for all such countries precise data were used). Detail classification and explanation of assigned climate zones per country can be found in Supplementary Table S4. The data for only one age group, six to 14, were used, while the data for OW and OB were analyzed separately. Effect size was pooled from all eligible studies using the DerSimonian–Laird Random effects model (REM) for meta-analysis (33). This REM model was preferred to a fixed effect model, since it is based on the assumption that a distribution of effects exists, resulting in heterogeneity among the study results. The heterogeneity of the results across studies was evaluated with the *I*^2^ statistic (34). The Mantel–Haenszel fixed-effects method was used when *I*^2^ was less than 50% (17).

### 2.7. Meta regression

Random-effects meta-regression analyses were used to evaluate whether the prevalence estimates differed according to region and country over time at two-time intervals, according to time differences of prevalence trends across 18 countries where data were available for both decades, by gender, and for OW and for OB separately, by latitudes, crossed over with climate zones. The same was done for the prevalence trends. Global warming after 2000, across all climate zones, was assumed according to findings (18). The significance value of the pooled effect size was estimated based on a 95% confidence interval (CI). Two-sided *p*-values of *p* = 0.05 or less were significant and a *Z* distribution was used in the meta regression.

## 3. Results

Of the 3,872 studies, through elimination and selection based on inclusion and exclusion criteria, 114 studies were selected (15, 35–147) (Figure 1).

Six of them (15, 36, 38, 40, 42, 77) included data from more than one country. The data from one measurement were classified into two studies which presented results based on gender (119, 120). The data from six European regions are shown: Central, East, North, South, Southeast and West, from 39 countries: Albania, (2 studies), Austria (2), Belarus (2), Belgium (4), Bulgaria (2), Cyprus (2), Czech Republic (3), Denmark (2), England (8), Finland (2), France (5), Germany (6), Greece (10), Greenland (2), Hungary (2), Iceland (1), Ireland (5), Italy (9), Latvia (2), Lithuania (3), Malta (2), Moldova (1), Montenegro (3), Netherlands (3), North Macedonia (2), Norway (4), Poland (3), Portugal (11), Romania (2), Russia (2), Serbia (2), Slovakia (2), Slovenia (2), Spain (10), Sweden (7), Switzerland (4), Turkey (8), and Ukraine (3). For detailed information of number of studies per countries and regions see (Supplementary Figure S1).

A total of 985,971 (boys/girls 499,071–486,900) subjects of an elementary school age, 6–14, made up the sample. The samples ranged from 255 to 133,156 subjects.

### 3.1. Study quality

The quality evaluation indicated 82 studies with a low risk of bias, 25 with a moderate, and only seven studies with a high-level risk of bias. For a detailed overview see (Supplementary Table S5).

### 3.2. By gender

#### 3.2.1. Overweight

The total prevalence of OB elementary school children aged six to 14 in Europe from the beginning of 2000 to 2020 is 17.55 (95% CI: 16.91–18.20). There is no gender difference, even though girls had lower values 17.39 (95% CI: 16.50–18.31) compared to boys 17.68 (95% CI: 16.69–18.71, *p* = 0.63). Based on study quality, the results are similar: low risk for girls 17.92 (95% CI: 16.89–19.01) compared to boys 18.04 (95% CI: 16.90–19.23).

#### 3.2.2. Obesity

The total prevalence of OB children is 5.56 (95% CI: 5.25–5.88), and the difference is in favor of the girls who have less obesity 4.78 (95% CI: 3.85–5.93) than the boys 5.76 (95% CI: 5.11–6.48, *p* = 0.03). Based on study quality, the results are similar with an added difference in favor of girls where low risk was noted 5.51 (95% CI: 5.02–6.06) when compared to boys 6.01 (95% CI: 5.45–6.62). See (Supplementary Table S5) for more details.

#### 3.2.3. First and second decade

There is a small difference in the prevalence of OW in the first decade: 17.13 (95% CI: 16.14–18.18) for girls, compared to 17.34 (95% CI: 16.33–18.40) for boys, while during the second decade it was 17.85 (95% CI: 16.23–19.58), and 18.32 (95% CI: 16.67–20.09), respectively. In the first decade the ratio of prevalence of OB for girls was 4.76 (95% CI: 4.29–5.29), compared to boys 5.26 (95% CI: 4.74–5.83), while during the second decade it was 5.96 (95% CI: 5.22–6.79) and 6.94 (95% CI: 6.08–7.89), respectively.

### 3.3. By region

#### 3.3.1. Overweight

**Figure 2:**
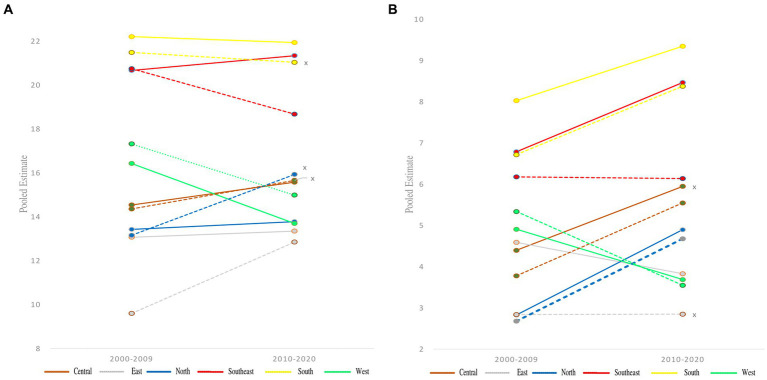
Pooled estimate for the prevalence over two time points, 2000–2009 to 2010–2020, by gender **(A)** overweight and **(B)** obesity in children aged 6–14 years across European regions according to IOTF definition criteria. Full line represent boys and line with dots girls, no significance for prevalence differences between two decades are marked with an x on the right side.

From the beginning of 2000 to 2020, across regions, girls showed the greatest prevalence of OW: it was highest for girls in South Europe 21.27 (95% CI: 19.81–22.80), and lowest in East Europe 11.61 (95% CI: 9.64–13.92). For boys the results were highest in South Europe 22.08 (95% CI: 20.58–23.65), and lowest in East Europe 13.24 (95% CI: 11.05–15.79), (Figures 2A,B, and overall results in Supplementary Figures S3A,B).

#### 3.3.2. Obesity

**Figure 3:**
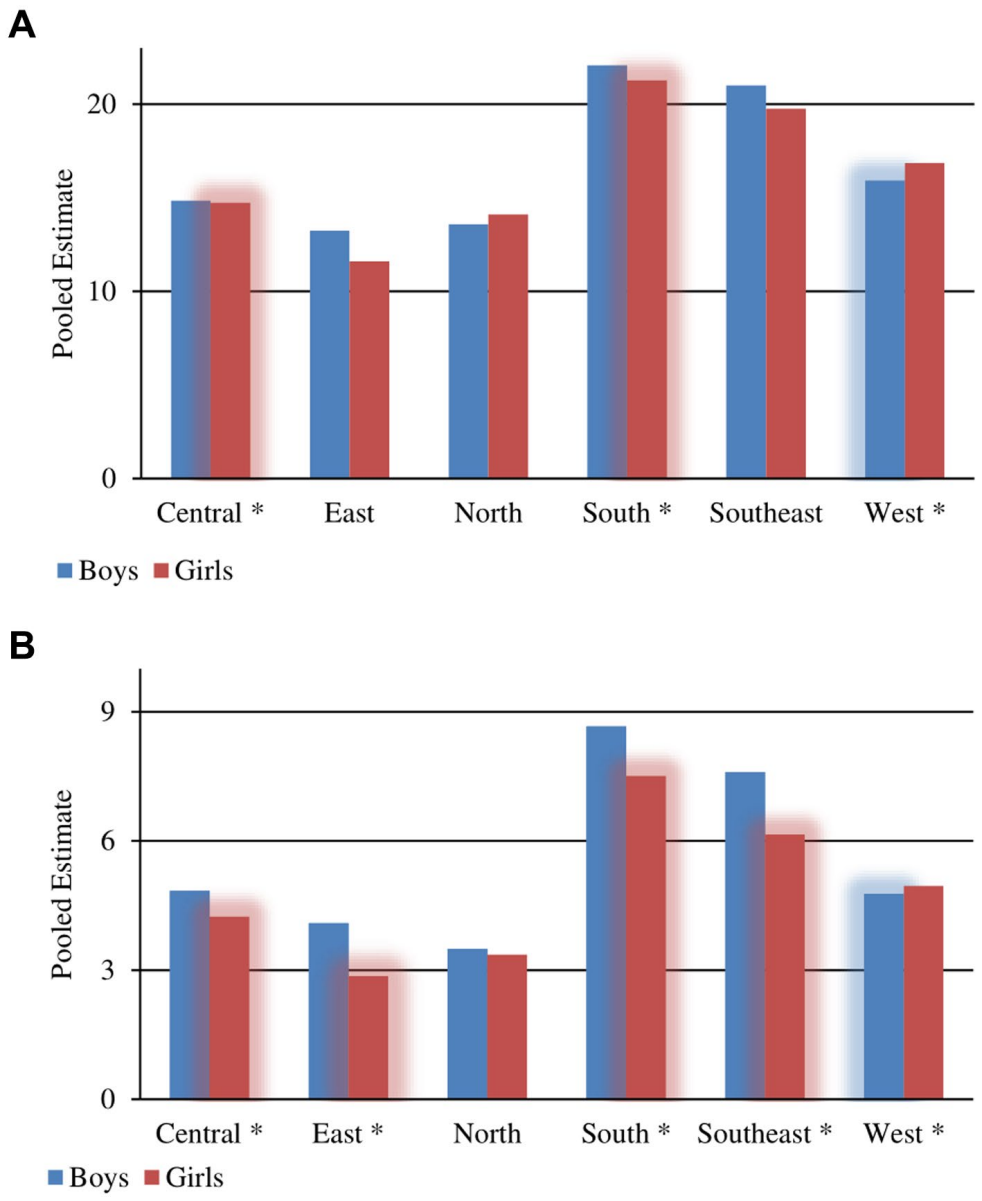
Pooled estimate for the prevalence from 2000 to 2020 by gender of **(A)** overweight and **(B)** obesity in children aged 6–14 years across European regions according to IOTF definition criteria. Glowing and ^*^ are presenting statistical significance of overall prevalence differences between gender.

Similar results were noted for OB in South Europe 7.51 (95% CI: 6.61–8.51), and the lowest in East Europe 2.86 (95% CI: 2.63–3.12). For boys the greatest prevalence was also noted in South Europe 8.66 (95% CI: 7.68–9.74), but the lowest was in North Europe 3.49 (95% CI: 2.90–4.19), (Figures 2A,B, and overall results in Supplementary Figures S3A,B). For more detailed data and significant differences across genders and through the two decades (see Supplementary Table S6) (see Figure 3).

### 3.4. By country

#### 3.4.1. Overweight

**Figure 4:**
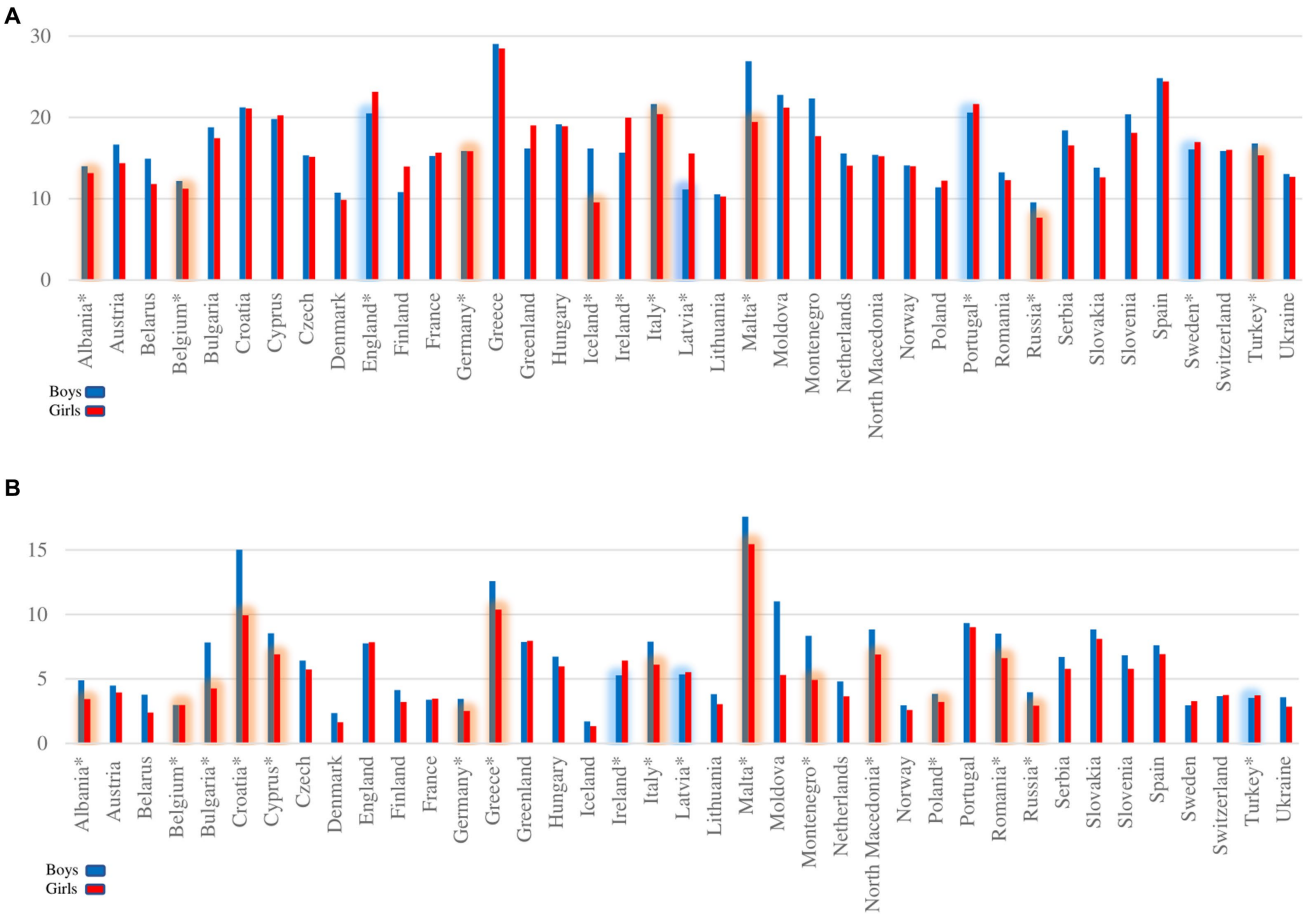
Overall pooled estimate for prevalence of **(A)** overweight and **(B)** obesity in children aged 6–14 years across 39 European countries according to IOTF definition criteria. Glowing and ^*^ are presenting statistical significance of overall prevalence differences between gender.

The greatest prevalence of OW among boys was noted in Greece 29.03 (95% CI: 26.32–31.90), and the lowest in Russia 9.55 (95% CI: 7.21–12.54), while for the girls a similar pattern was noted, with lower values: Greece 28.49 (95% CI: 25.80–31.33) and Russia 7.67 (95% CI: 5.73–10.19), respectively, (see Supplementary Table S7 and Figures 4A,B).

#### 3.4.2. Obesity

For boys, the greatest prevalence was noted in Malta 17.59 (95% CI: 11.82–25.36), and the lowest in Iceland 1.70 (95% CI: 0.76–3.74), while for girls we find the same pattern but with lower values: Malta 15.45 (95% CI: 9.49–24.18) and Iceland 1.33 (95% CI: 0.55–3.15), respectively, (see Supplementary Table S7 and Figures 4A,B). A more detailed significant difference in prevalence across countries over time, as well as gender differences, are both shown in the Supplementary Figures S2A,B, S3A,B.

### 3.5. By climate zone

#### 3.5.1. Overweight

**Figure 5:**
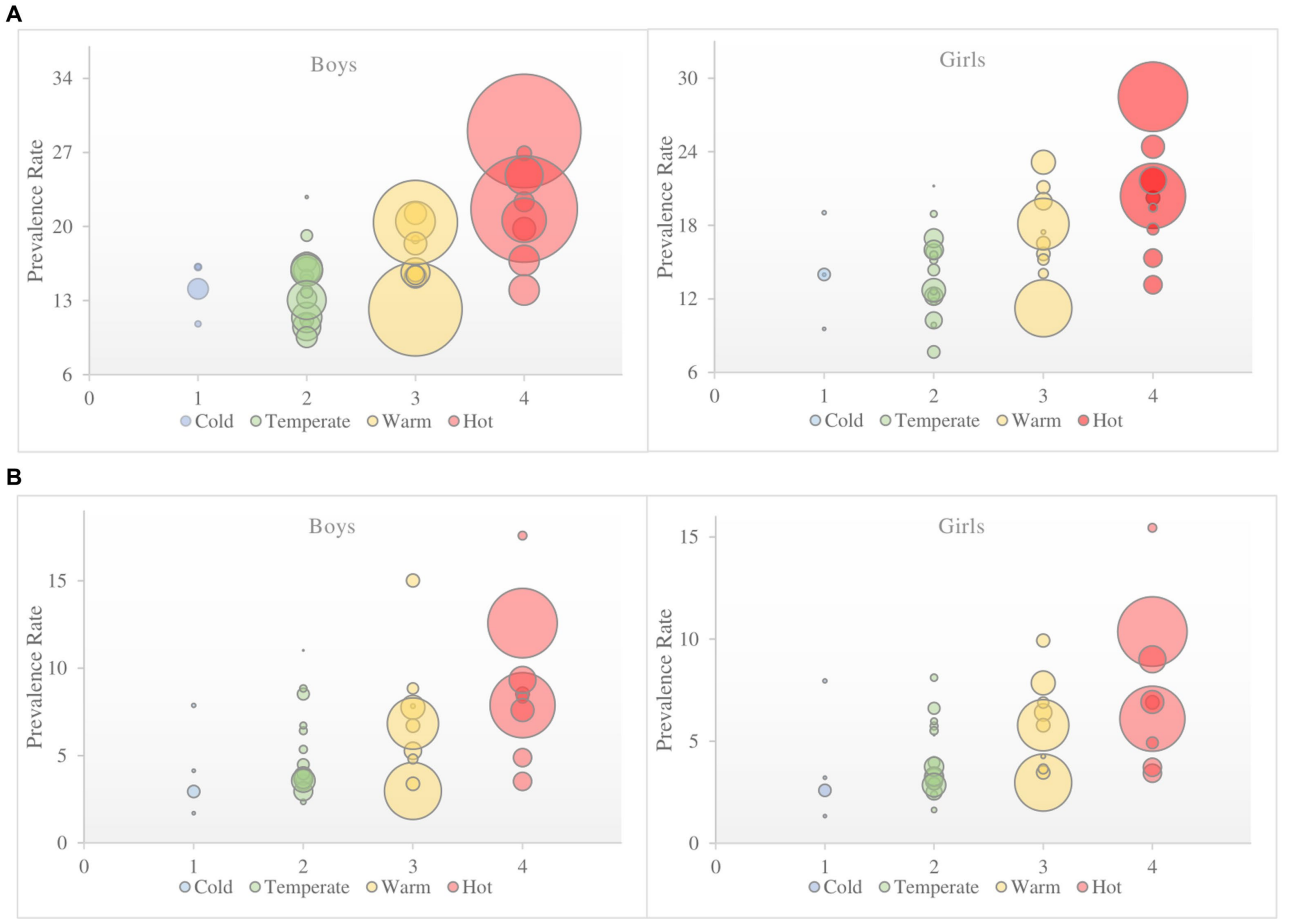
The overall pooled estimate for prevalence from European countries by Köppen–Geiger climate zones during 2000–2020 of **(A)** overweight and **(B)** obesity in children aged 6–14 years according to IOTF definition criteria. Size of the circles that represent countries are defined by subject sample number.

From the beginning of 2000 to 2020, according to the Köppen–Geiger climate zones, the greatest prevalence of OW among girls was in the hot zone 21.31 (95% CI: 20.19–22.43), compared to boys 22.20 (95% CI: 21.29–23.14). The lowest scores were recorded in the temperate zone 13.92 (95% CI: 12.62–15.23) for girls, and 14.03 (95% CI: 12.69–15.42) for boys, (Figures 5A,B).

#### 3.5.2. Obesity

For OB, the greatest prevalence was noted for girls in the hot zone 7.02 (95% CI: 6.30–7.82). Progressively lower scores were recorded with a decrease in temperature, ending with the results for the cold zone 3.28 (95% CI: 2.17–4.92). These results are lower compared to those of the boys, but they still follow the same pattern: highest in the hot zone 8.23 (95% CI: 7.55–8.96) and lowest in the cold zone 3.58 (95% CI: 2.39–5.33), (Figures 5A,B). For a more detailed overview of prevalence over time and gender differences across Köppen–Geiger climate zones of Europe see (Supplementary Table S8 and Supplementary Figures S3A,B, S4A,B).

### 3.6. Meta regression

**Figure 6:**
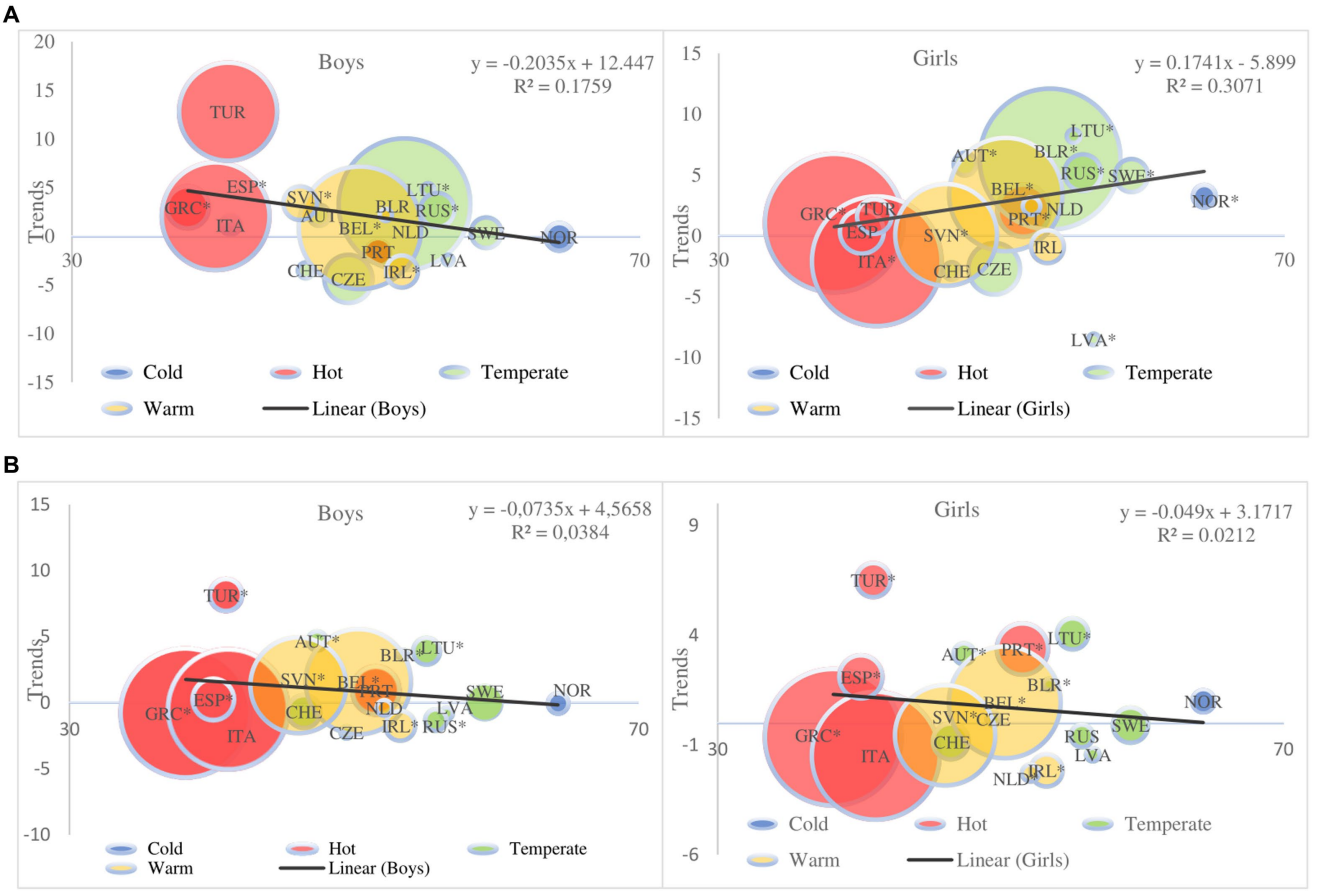
Meta-regression plot of trends by gender **(A)** overweight and **(B)** obesity. Meta-regression plot shows 18 European countries trends and differences by Köppen–Geiger climate zones during 2000–2020 according to latitude and with the same degree of equal warming from findings in (18) of **(A)** overweight and **(B)** obesity in children aged 6–14 years according to IOTF definition criteria. Solid lines indicate regression, ^*^statistical significance. Size of the circles that represent countries are defined by subject sample number; size of the circles that represent countries are defined by subject sample number.

By monitoring the state in 18 countries for which data was provided for both decades, a meta regression was used to determine the impact of global warming based on climate zones (Figures 6A,B). A significant impact of global warming through climate zones was determined on the prevalence of OW and OB. Eta = 0.22 for OW, and Eta = 0.52 for OB. There are no significant gender differences between the decades, nor is there any significant interaction. No significant impact of climate zones was determined on the trend of growth across genders for OW and OB. A significant difference was noted for prevalence across climate zones: hot and temperate, warm and cold, and warm and temperate for OW, and hot and temperate and cold, warm and temperate for OB. For OW it was Eta = 0.38, and for OB Eta = 0.23.

## 4. Discussion

The analyses provide insight into the prevalence of OW and OB among elementary school children in almost all of Europe. The details from 114 studies, most with a low risk of bias, are presented. Only data from the IOTF cut-off points are presented, which together offer a clear image of the impact of global warming after 2000 (18). Even though Europe is divided into four Köppen–Geiger climate zones, it is possible to make an initial assessment and take action against the lack of detailed monitoring of the state of global warming over the past 20 years for each country individually. These data provide a crude image, but still the first of its kind, of the prevalence of OB among children, which is significantly related to the warming of all the regions of Europe. Even though using the WHO cut-offs provides a higher value of the prevalence rates compared to the IOTF cut-offs and CDC (12, 13, 28), the results obtained from the IOTF data in Europe are alarming. A more recent approach to the problem of the greater prevalence of OB in the warmer parts of Europe is the report of three mutually connected pandemics: obesity, undernutrition, and climate change (19), and the impact of global warming across climate zones. Our study is the first one to take a step towards explaining the problem of this prevalence.

The complex problem of weight status and obesity are connected to major factors such as, physical activity, climate change-global warming, income, diet, and green space (20, 23, 148).

While global warming is a relatively new topic, there are a few studies and reports that show mixed data on the effects on weight status, but there is clear evidence that warner climates negatively affect weight status and obesity rates (20, 23, 149). Net of sex, age, race, education, earnings, neighborhood characteristics, and physical activities, individuals living in warmer climates have higher BMI and weight, and are more likely to be overweight and obese than those living in colder climates. The effect of atmospheric temperature on obesity, as a multi influenced factor which affects many aspects of everyday life, is stronger than the effect of most physical activities (23). Limitations of the study (23) and possible explanation of results that atmospheric temperature has stronger effects then most physical activity lie in the collecting of data by questionnaire and the possible biased answers of the subjects.

A larger global warming impact of noticeable temperature increase by 1.5°C can be seen in the cold zone compared to hot or warm zone, that already have higher average temperatures. Confirmation can be found in the results of the largest increase in OW between two decades of 5.31% for girls and 1.81% for boys, and for OB 2.8% for girls and 1.53% for boys, respectively, as well as in the North region (mostly predominantly with a cold and temperate climate) of 2 and 2.07% for girls and boys, respectively. Why girls showed a higher rise compared to boys in only the cold zone, or why there is a gender differences are still unknow, while the results continue to show that they are still significantly better than boys. That rise only continued the alarming state of the hot and warm zones which also showed a positive trend of 1.49% and 1.37% for girls and boys, respectively. A possible explanation for this is adaptive nature that forces people to find shelter in cool indoors with air conditioning and spend more time in sedentary behavior, snacking, and less time physical active, therefore contributing to the obesity epidemic (148). Since adults and children are most active between 15°C and 20°C, residents of warm climates are most active in the fall and winter, residents of cold climates are most active in spring and summer, and residents of temperate climates can be active year-round (148, 150). However, Kanazawa (23), stated that compared to geographic relocation, global warming is expected to have a much smaller effect on obesity. The limitation of his proposition is that it is based on an assumption dating back from 2017 (151), and the estimation that an increase of 5.6 C from 1961 to 2081 will lead to an increase in weight of 1 kg. Due to many factors that affect the complexity of the obesity problem and speed of change, there is need for more studies to predict and outline more precise development.

The results of this meta-analysis add to the state of OB worldwide from 1980 to 2015, which showed a double increase in OB in 70 countries (3). Since all age categories were studied, a greater rate of increase among children was noted compared to adolescents and older populations. Only data from the IOTF definition of cut-off points were used (3). Confirmation was found in (12), which analyzed the results of 323,648 boys and 313,285 girls from 21 European countries, ages six to nine. These findings also confirmed the greatest prevalence of OB to be in the South of Europe, with Sweden and Moldavia showing the best results, unlike Malta.

Data from 15 European countries, with the exclusion of Malta and Cypress (152), led to the conclusion that OW children are more likely to become OB in the South of Europe compared to children in West Europe, which can be confirm in results in the country with the lowest values of OB, Ireland with 5.9, while Greece had the most alarming results of 14.6 for children under nine.

From 2001 to 2010, a group of researchers studied Greece, a country in the South of Europe, more closely, also based on IOTF data. Their meta-analysis included 25 studies of 219,996 boys and 210,772 girls aged one to 12. They concluded that from 2001 to 2010, 3/10 were OW, while 1/10 were OB. Boys were more OW, 24.1%, compared girls, 23.2%, and more obese, 11%, compared to girls, 9.7%. The rising trend for both genders lasted from 2001 to 2003, with a stabilization period from 2003 to 2010 (16).

From 1999 to 2016, the meta-analysis in (17) noted a stabilization in the rise of OB among children in Europe, with a difference between countries, a finding similar to our own. Even though they used different geographical names for the regions of Europe, our divisions matched. The authors cited that the greatest prevalence of OB among children aged seven to 13 based on the IOTF cut-off values was in the Mediterranean 10.1% and then the Iberian region 8.8%, while the Central region 3.2% had the lowest prevalence. A slight difference in the results, which followed the same pattern, can be seen in our study. The greatest prevalence of OB is in South Europe: boys 8.66% and girls 7.51%, and in South–East Europe: boys 7.60% and girls 6.15%. The lowest prevalence among girls was noted in East Europe 2.86%, and among boys in North Europe 3.49%. Our results show mixed trends for gender differences only in the East and Southeast region. However, the answer is unclear, as there are differences in the number of studies and countries between the two decades (see Supplementary Table S6). West region showed a decreasing trend for both genders, possibly because those countries have the highest income of all European countries (153), more awareness among officials regarding global warming and obesity problems and therefore projects for preventing them in the form of diet programs (154) and stricter physical activity regimes in schools (155). The prevalence across countries is different. Italy has the greatest values for prevalence of OB among boys 15.3% and girls 14.9% aged seven to 13, as does Malta: 14.8% and 13.5% respectively, while Switzerland had the lowest prevalence among boys 2.1% and girls 2.6%. However, in our study, the prevalence of OB among boys was greatest in Malta 17.59%, and lowest in Iceland 1.70%, while the same prevalence was noted for girls but with lower values: Malta 15.45% and Iceland 1.33%, respectively (17).

To understand the complexity of the global problem of OB, it is necessary to carry out detailed analyses of all the factors on both a smaller and larger sample of subjects, which would include numerous factors: both those which contribute to OB and those which are successfully used in its prevention. Studies which focused on the level of physical activity, which has the greatest correlation with OB, can provide one part of the answer (156, 157). It is not possible to get a definitive response on the emergence of OB among children from the results provided by one study (156). Researchers studied levels of physical activity among children 11 to 15, primarily in European countries (*n* = 32) from 2002 to 2010, and reached the conclusion that most children do not reach the prescribed level of moderate to high intensity exercise for a period of 60 min. Even though a significant increase in the level of physical activity among boys from 16 countries was noted from 2002 to 2010 (17.0 and 18.6%, *p* = 0.05), nine countries showed a decrease. Girls from 10 countries showed an increase, but 8 countries showed a decrease in physical activity, which led to the conclusion that girls are less successful in reaching the recommended level of physical activity. Even though a rising trend of participation in physical activity among boys was confirmed (157), this does not explain the greater overall prevalence of OB among boys compared to girls in Europe following 2000. The only confirmation of OB and the achieved level of physical activity can be found in the results from countries with more rate of OB where accelerometers were used to measure children’s levels of physical activity. Meta regression results show larger OW and OB trends in the warmer climate zones then in cold ones. A larger impact of global warming of 1.5°C in cold climate can be seen in meta regression diagram only for OW girls but not in boys. This finding is confusing; even though there is just one country from the cold climate zone, that do not diminish the results that show that initial OW state of girls leads them into OB much faster than boys, due to global warming. That can be substantiated with results of (157), which shows low values of 559 min of daily physical activity were noted in South and South–East Europe, such as 492 min in Portugal and Greece, which corresponds to the greater prevalence of OB in these countries. Higher levels of physical activity were noted in the countries of North Europe, such as Norway with 804 min, which have a lower prevalence of OB, that also matches our findings but not for OW girls.

## 5. Limitations

This meta-analysis is not exempt from the usual limitations, such as the differences in quality of the studies included, that is, the limited nature of some of the data. In addition, the first specific limitation is that some studies relied on non-representative samples, which could jeopardize the validity of the evaluation of the prevalence rate. The second is the use of referential cut-off points prescribed by the IOTF (27, 28), and the inability to compare the results with those of other studies. The third are the smaller differences between two cut-off points which still had an impact on prevalence estimates. The fourth is the lack of precise data pertaining to the location of subject selection in some studies, which made deviations in determining the precise climate image of countries with two or more climate zones possible. The fifth one is the differences in the features of the samples themselves, the number of studies originating from a country, and the quality of studies which increased the heterogeneity of some studies and decreased the quality of the data. And the precision of the impact of global warming on the prevalence of OB is affected by the lack of data on the progression of temperature growth on an annual basis, especially in each country which limits obtained results.

## 6. Conclusion

The current physical activity levels in schools are insufficient for fighting the obesity problem which now has one more, significant contributor in the form of global warming that has an impact on the prevalence of OW and OB among children in all the climate zones of Europe, especially the hot zone. It is not known with certainty to what extent the increasing trend will grow, or whether the decreasing trend in some countries and regions will remain unchanged, but we can conclude that gender differences for OB will disappear if further preventive methods are not implemented immediately. We recommend a stricter dietary regimen and increased physical activity in schools. For future research, it is necessary to monitor the level of physical activity with precise changes in temperature by country, as well as sedentary time with the dietary regimen in schools in order to determine the accurate impact ratio between the aforementioned factors.

## Data availability statement

The original contributions presented in the study are included in the article/Supplementary material, further inquiries can be directed to the corresponding author.

## Author contributions

NP was the leader of the research group that conducted the study. NP and SP were designed the methodology approach of study and wrote the first draft of the manuscript. NP, MD, and SP collected the data, rechecked data accuracy, and contributed in conceptualization. NP and MD led the statistical analysis and visualization. SP and MD were in charge of supervision. All authors contributed to the article and approved the submitted version.

## Conflict of interest

The authors declare that the research was conducted in the absence of any commercial or financial relationships that could be construed as a potential conflict of interest.

## Publisher’s note

All claims expressed in this article are solely those of the authors and do not necessarily represent those of their affiliated organizations, or those of the publisher, the editors and the reviewers. Any product that may be evaluated in this article, or claim that may be made by its manufacturer, is not guaranteed or endorsed by the publisher.

## Footnotes

^1^ www.latitude.to
