# Supplementary material for: Gender differences and climate zones in overweight and obesity prevalence in European elementary school children from 2000 to 2020: a systematic review and meta-analysis
Source: Front Public Health. 2023 Sep 21;11:1198877. doi: 10.3389/fpubh.2023.1198877 (PMC10558048; doi:10.3389/fpubh.2023.1198877)
Supplement: Supplementary file 1 [file Table_1.PDF]

Supplementary Material

Gender differences and climate zones in overweight and obesity prevalence in European elementary school children from 2000 to 2020: A systematic review and meta–analysis

Nikola Prvulović*, Miodrag Djordjević, Saša Pantelić

*** Correspondence:** Ph.D. Candidate Nikola Prvulović: nikolaprvulovic87@gmail.com

Supplementary material

Table S1. Search strategy

Table S2. Characteristics of cross–sectional, cut-off points by IOTF, included studies

Table S3. Quality assessment of prevalence studies following the the Joanna Briggs Institute tool

Table S4. Number of Koppen-Geiger climate zones per country

Table S5. Prevalence from 2000 to 2020 and overall (pool estimate (95% confidence interval) of obesity and overweight in European children aged 6–14 years by quality of included studies

Table S6. Prevalence by European regions through 2000–2020 and overall (pool estimate (95% confidence interval) of obesity and overweight in European children aged 6–14 years of included studies

Table S7. Pooled estimate and prevalence trends of overweight and obesity in elementary school children aged 6–14 years across 39 European countries using IOTF definition criteria

Table S8. Prevalence by Koppen–Geiger climate zones through 2000–2020 and overall (pool estimate (95% confidence interval) of obesity and overweight in European children aged 6–14 years of included studies

Figure S1. European map of the number of studies per country and region

Figure S2. Pooled estimate for the prevalence from 2000 to 2020 by gender of (A) overweight and (B) obesity in children aged 6–14 years across 18 European countries according to IOTF defnition criteria

Figure S3. Pooled estimate for the prevalence accros Koppen–Geiger climate zones by gender of (A) overweight and (B) obesity in children aged 6–14 years across European countries according to IOTF defnition criteria

Figure S4. Pooled estimate for the prevalence from 2000 to 2020 by gender of (A) overweight and (B) obesity in children aged 6–14 years across 4 Koppen–Geiger climate zones according to IOTF defnition criteria

This supplementary material has been provided by the authors to give readers additional data and information about their work.

Table S1. **Search strategy**

Search terms			
**Population**	**Outcome**	**Study design, type of studies**	**Location**
children, childhood, schoolchildren, adolescent, school–aged	obesity, overweight, body composition, weight status	prevalence, trend, epidemiolog(), cross–sectional*, observat()	Albania „ALB“, Austria „AUT“, Belarus „BLR“, Belgium „BEL“, Bulgaria „BGR“, Croatia „HRV“, Cyprus „CYP“, Czech Republic „CZE“, Denmark „DNK“, England „ENG“, Finland „FIN“, France „FRA“, Germany „DEU“, Greece „GRC“, Greenland „GRL“, Hungary „HUN“, Iceland „ISL“, Ireland „IRL“, Italy „ITA“, Latvia „LVA“, Lithuania „LTU“, Malta „MLT“, Moldova „MDA“, Montenegro „MNE“, Netherlands „NLD“, North Macedonia „MKD“, Norway „NOR“, Poland „POL“, Portugal „PRT“, Romania „ROU“, Russia „RUS“, Serbia „SRB“, Slovakia „SVK“, Slovenia „SVN“, Spain „ESP“, Sweden „SWE“, Switzerland „CHE“, Turkey „TUR“, Ukraine „UKR“.

*, – Or; () – All possible variations of word endings are included; *–Cross sectional analysis at retrospectional or prospective cohort study (one time point); „ “– The country codes refer to the International Organization for Standardization ISO 3166–1, Alpha–3 country codes and countries were listed in alphabetical order by their full names.*

Table S2. Characteristics of cross–sectional, cut-off points by IOTF, included studies

First author, reference number	Survey period	Subject characteristic		N by gender		Results						Region	Country	Latitude	Climate zone
						Together		Boys		Girls					
		Age	N	Boys (%)	Girls (%)	Ob (%)	Ow (%)	Ob (%)	Ow (%)	Ob (%)	Ow (%)				
Hyska, [35]	2013	7–10	5810	2993 (51.51)	2817 (48.49)	193 (3.32)	646 (11.11)	115 (3.84)	331 (11.06)	78 (2.77)	315 (11.18)	SE	Albania	41.09	Csa
Trap, [36]	2013–2015	10.9±2.6	9799	4900 (50)	4899 (50)	505 (5.15)	1602 (16.34)	299 (6.1)	852 (17.38)	206 (4.2)	750 (15.31)	SE	Albania	41.09	Csa
Mayer, [37]	2009–2011	6–12	5446	2764 (50.75)	2682 (49.25)	338 (6.2)	931 (17.09)	188 (6.8)	470 (17)	150 (5.6)	461 (17.2)	C	Austria	47.41	Dfb
Yngve, [15]	2003	10.9±0.6	1222	566 (46.31)	656 (53.68)	28 (2.29)	158 (12.92)	12 (2.12)	84 (14.84)	16 (2.44)	74 (11.28)	C	Austria	47.41	Dfb
Klimatskaya, [38]	2010	11	1000	502 (50.2)	498 (49.8)	46 (4.6)	160 (16)	29 (5.77)	83 (16.53)	17 (3.41)	77 (15.46)	E	Belarus	53.43	Dfb
Marfina, [39]	2000	8–13	1486	798 (53.7)	688 (46.3)	28 (1.88)	164 (11.03)	15 (1.88)	92 (11.52)	13 (1.89)	72 (10.46)	E	Bealrus	53.43	Dfb
Brug, [40]	2010	10–12	1003	481 (47.96)	522 (52.04)	30 (3)	151 (15.05)	18 (3.7)	81 (16.9)	12 (2.3)	70 (13.5)	W	Belgium	50.3	Cfb
Roelants, [41]	2002–2004	6–14	7547	3743 (49.6)	3804 (50.4)	211 (2.8)	981 (13)	112 (3)	532 (14.2)	99 (2.6)	449 (11.8)	W	Belgium	50.3	Cfb
Wijnhoven, [42]	2007–2010	6–9	133156	67783 (50.9)	65373 (49.1)	5665 (4.25)	15064 (11.31)	2492 (3.67)	6765 (9.98)	3173 (4.85)	8299 (12.69)	W	Belgium	50.3	Cfb
Yngve, [15]	2003	11.5±0.5	987	508 (51.46)	479 (48.53)	13 (1.32)	75 (7.64)	3 (0.6)	45 (8.86)	10 (2.08)	30 (6.26)	W	Belgium	50.3	Cfb
Mladenova, [43]	2012–2014	8–14	807	402 (49.8)	405 (50.2)	47 (5.82)	144 (17.84)	31 (7.7)	75 (18.65)	16 (3.95)	69 (17.03)	SE	Bulgaria	42.43	Cfb
Mladenova, [44]	2012–2014	8–11	468	237 (50.64)	231 (49.36)	30 (6.41)	87 (18.58)	19 (8.01)	45 (18.99)	11 (4.76)	42 (18.18)	SE	Bulgaria	42.43	Cfb
Milanovic, [45]	2015	8–9	2738	1369 (50)	1369 (50)	383 (13.98)	573 (20.92)	236 (17.23)	295 (21.54)	147 (10.73)	278 (20.3)	S	Croatia	44.28	Cfb
Milanovic, [46]	2015	8.62±0.6	5591	2811(50.27)	2780 (49.73)	624 (11.16)	1191 (21.3)	368 (13.09)	593 (21.09)	256 (9.2)	598 (21.51)	S	Croatia	44.28	Cfb
Savva, [47]	1999–2000	6–14	1870	907 (48.5)	963 (51.5)	130 (6.95)	343 (18.34)	67 (7.38)	164 (18.08)	63 (6.54)	179 (18.58)	SE	Cyprus	35.1	Csa
Savva, [48]	1997–2003	11.5±0.2	7060	3618 (51.24)	3442 (48.76)	559 (7.9)	1525 (21.6)	318 (8.8)	774 (21.4)	241 (7.0)	751 (21.8)	SE	Cyprus	35.1	Csa
Kunesova, [49]	2005	6–12	470	257 (54.7)	213 (45.3)	52 (11.06)	93 (19.78)	31(12.06)	55 (21.4)	21 (9.86)	38 (17.84)	C	Czech	49.48	Dfb
Kunešová, [50*]	2008	7	1531	778 (50.8)	753 (49.2)	59 (3.85)	170 (11.1)	34 (4.37)	89 (11.44)	25 (3.32)	81 (10.75)	C	Czech	49.48	Dfb
Wijnhoven, [42]	2007–2010	7	1271	638 (50.2)	633 (49.8)	69 (5.4)	165 (13)	32 (5)	80 (12.5)	37 (5.9)	85 (13.4)	C	Czech	49.48	Dfb
Matthiessen, [51]	2000–2002	7–14	636	332 (52.2)	304 (47.8)	20 (3.14)	76 (11.9)	11 (3.31)	34 (10.24)	9 (2.96)	42 (13.81)	N	Denmark	55.56	Dfb
Yngve, [15]	2003	11.3±0.4	1109	561 (50.58)	548 (49.42)	11 (1.0)	100 (9.01)	9 (1.6)	62 (11.05)	2 (0.36)	38 (6.93)	N	Denmark	55.56	Dfb
Basterfield, [52*]	2006–2007	6–8	425	211 (49.6)	214 (50.4)	27 (6.4)	75 (17.6)	15 (7.1)	35 (16.6)	12 (5.6)	40 (18.7)	W	England	53.55	Cfb
Dummer, [53]	1998–2003	9–10	15416	7902 (51.25)	7514 (48.75)	847 (5.5)	2712 (17.6)	419 (5.3)	1209 (15.3)	428 (5.7)	1503 (20)	W	England	53.55	Cfb
Harding, [54]	2002–2003	11–13	5515	2920 (52.94)	2595 (47.06)	453 (8.21)	1172 (21.25)	239 (8.18)	558 (19.1)	214 (8.24)	614 (23.67)	W	England	53.55	Cfb
Hughes, [55]	2005	8.3±2.0	1548	797 (51.48)	751 (48.52)	295 (19.05)	486 (31.4)	169 (21.2)	258 (32.37)	126 (16.77)	228 (30.35)	W	England	53.55	Cfb
Stamatakis, [56]	1996; 2002–2003	8–10	1844	951 (51.6)	893 (48.4)	132 (7.15)	492 (26.68)	64 (6.73)	250 (26.28)	68 (7.61)	242 (27.1)	W	England	53.55	Cfb
Steele, [57]	2007	10.3±0.03	1862	820 (44)	1042 (56)	94 (5.04)	332 (17.83)	32 (3.9)	130 (15.9)	62 (6)	202 (19.4)	W	England	53.55	Cfb
Eloranta, [58]	2007–2009	6–8	510	263 (51.57)	247 (48.43)	28 (5.5)	44 (8.62)	12 (4.6)	17 (6.5)	11 (4.5)	27 (10.9)	N	Finnland	62.53	Dfc
Stigman, [59]	2009	8.6±0.1	304	153 (50.3)	151 (49.7)	6 (2)	52 (17.1)	5 (3.3)	25 (16.3)	1 (0.7)	27 (17.9)	N	Finnland	62.53	Dfc
Rolland–Cachera, [60]	2000	7–9	1582	786 (49.7)	796 (50.3)	58 (3.66)	229 (14.47)	28 (3.6)	115 (14.7)	30 (3.8)	114 (14.3)	W	France	46.42	Cfb
Salanava, [61]	2007	7–9	1014	512 (50.5)	502 (49.5)	28 (2.76)	133 (13.11)	14 (2.8)	57 (11.3)	14 (2.8)	76 (14.9)	W	France	46.42	Cfb
Klein–Platat, [62]	2001	12.1±0.6	4323	2223 (51.4)	2103 (48.6)	227 (5.25)	985 (22.78)	124 (5.57)	505 (22.71)	103 (4.9)	480 (22.82)	W	France	46.42	Cfb
Heude, [63]	1992; 2000	6–12	552	276 (50)	276 (50)	17 (3.07)	69 (12.5)	4 (1.44)	27 (9.78)	13 (4.71)	42 (15.21)	W	France	46.42	Cfb

First author, reference number	Survey period	Subject characteristic		N by gender		Results						Region	Country	Latitude	Climate zone
						Together		Boys		Girls					
		Age	N	Boys (%)	Girls (%)	Ob (%)	Ow (%)	Ob (%)	Ow (%)	Ob (%)	Ow (%)				
Thibault [64]	2004–2005	11–14	1292	672 (52.)	620 (48)	28 (2.2)	177 (13.7)	18 (2.67)	110 (16.36)	10 (1.61)	67 (10.8)	W	France	46.42	Cfb
Bluher, [65]	2000–2004; 2008	8–12	8981	4754 (52.9)	4227 (47.1)	427 (4.75)	2030 (22.6)	322 (6.77)	1208 (25.41)	105 (2.48)	822 (19.45)	C	Germany	51.09	Dfb
Kromeyer–Hauschild, [66]	2001	7–14	1915	964 (50.3)	915 (49.7)	26 (1.35)	217 (11.33)	17 (1.76)	111 (11.51)	9 (0.98)	106 (11.58)	C	Germany	51.09	Dfb
Nagel, [67]	2006	7.6±0.4	1063	565 (53.15)	498 (46.85)	38 (3.6)	179 (16.8)	20 (3.5)	93 (16.5)	18 (3.6)	86 (17.3)	C	Germany	51.09	Dfb
Will, [68]	2002	6–7	525	274 (52.2)	251 (47.8)	13 (2.47)	64 (12.2)	3 (1.09)	24 (8.76)	10 (3.98)	38 (15.13)	C	Germany	51.09	Dfb
Angelopoul, [69]	2003–2004	11	312	153 (49)	159 (51)	30 (9.6)	107 (34.3)	18 (11.8)	45 (29.4)	12 (7.5)	62 (39.0)	SE	Greece	38.16	Csa
Brug, [40]	2010	10–12	1091	503 (46.1)	588 (53.9)	113 (10.35)	445 (40.78)	56 (11.2)	223 (44.4)	57 (9.7)	222 (37.7)	SE	Greece	38.16	Csa
Grigorakis, [70]	2010–2011	9.9±1.1	124113	63064 (50.81)	61049 (49.19)	10938 (8.81)	30778 (24.8)	6054 (9.6)	15577 (24.7)	4884 (8)	15201 (24.9)	SE	Greece	38.16	Csa
Hassapidou, [71]	2006	9.98±0.95	276	138 (50)	138 (50)	31 (11.7)	77 (24.1)	16 (11.6)	36 (26.1)	15 (10.9)	31 (22.4)	SE	Greece	38.16	Csa
Manios, [72]	2005–2006	10–12	481	231 (48)	250 (52)	60 (12.47)	137 (28.48)	35 (15.15)	69 (29.9)	25 (10)	68 (27.2)	SE	Greece	38.16	Csa
Manios, [73]	2007	9–13	2492	1241 (49.8)	1251 (50.2)	285 (11.43)	771 (30.93)	165 (13.29)	391 (31.5)	120 (9.59)	380 (30.37)	SE	Greece	38.16	Csa
Papadimitriou, [74]	2003–2004	6–11	4131	2054 (49.72)	2077 (50.28)	457 (11.06)	1121 (27.13)	252 (12.3)	571 (27.8)	205 (9.9)	550 (26.5)	SE	Greece	38.16	Csa
Tambalis, [75]	1997– 2007	8–9	71227	36396(51.1)	34831 (48.9)	8341 (11.7)	18945 (26.6)	4440 (12.2)	9645 (26.5)	3901 (11.2)	9300 (26.70	SE	Greece	38.16	Csa
Tokmakidis, [76]	2006	8.9±1.6	709	381 (53.7)	328 (46.3)	105 (14.8)	183 (25.8)	61 (16.0)	96 (25.2)	44 (13.4)	87 (26.5)	SE	Greece	38.16	Csa
Wijnhoven, [42]	2007–2010	7–9	5269	2581 (49)	2688 (51)	754 (14.31)	1427 (27.08)	365 (14.14)	709 (27.46)	389 (14.47)	718 (26.7)	SE	Greece	38.16	Csa
Høyer, [77*]	2010–2012	5–9	528	286 (54.16)	242 (45.84)	49 (9.28)	103 (19.5)	29 (10.14)	57 (19.93)	20 (8.26)	46 (19)	N	Greenland	69.38	Et–Dfc
Rex, [78]	2011–2013	6.6±0.6	607	308 (50.74)	299 (49.26)	41 (6.75)	96 (15.81)	18 (5.84)	39 (12.66)	23 (7.69)	57 (19.06)	N	Greenland	69.38	Dfc
Brug, [40]	2010	10–12	1020	458 (44.9)	562 (55.1)	54 (5.29)	254 (24.9)	31 (6.8)	127 (27.7)	23 (4.1)	127 (22.6)	C	Hungary	47.09	Dfb
Wijnhoven, [42]	2007–2010	7	1235	553 (44.77)	682 (55.23)	93 (7.53)	174 (14.08)	37 (6.7)	67 (12.2)	56 (8.2)	107 (15.7)	C	Hungary	47.09	Dfb
Yngve, [15]	2003	11.3±0.3	729	352 (48.28)	377 (51.72)	11 (1.51)	93 (12.75)	6 (1.7)	57 (16.19)	5 (1.32)	36 (9.55)	N	Iceland	64.55	Dfc
Barron, [79]	2007	6–13	764	426 (55.75)	338 (44.25)	54 (7.06)	129 (16.88)	31 (7.27)	74 (17.37)	23 (6.8)	55 (16.27)	W	Ireland	53.25	Cfb
O'Neill, [80]	2003–2004	9–12	299	149 (49.84)	150 (50.16)	20 (6.68)	51 (17.05)	5 (3.4)	24 (16.1)	15 (10)	26 (17.3)	W	Ireland	53.25	Cfb
Watkins, [81]	1989–1991; 2000	12	1047	532 (50.82)	515 (49.18)	49 (4.68)	190 (18.14)	25 (4.7)	79 (14.8)	24 (4.7)	111 (21.6)	W	Ireland	53.25	Cfb
Whelton, [82]	2001–2002	6–14	10033	4953 (49.37)	5080 (50.63)	682 (6.8)	1876 (18.7)	304 (6.14)	848 (17.1)	378 (7.44)	1028 (20.23)	W	Ireland	53.25	Cfb
Wijnhoven, [42]	2007–2010	7–9	1986	1022 (51.46)	964 (48.54)	90 (4.53)	321 (16.16)	44 (4.3)	136 (13.3)	46 (4.77)	185 (19.19)	W	Ireland	53.25	Cfb
Albertini, [83]	2003–2005	6–9	5636	2873 (51)	2763 (49)	1053 (18.68)	504 (8.94)	265 (9.22)	505 (17.57)	239 (8.65)	548 (19.83)	S	Italia	41.17	Cfb
Baratta, [84]	1999–2001	11–14	42326	20801 (49.15)	21525 (50.85)	3193 (7.54)	9665 (22.83)	1964 (9.44)	5161 (24.8)	1229 (5.71)	4504 (20.9)	S	Italia	41.17	Csa
Bertoncello, [85]	2004	9–11	12832	6521 (50.8)	6311 (49.2)	712 (5.54)	2722 (21.21)	386 (5.91)	1373 (21.05)	326 (5.16)	1349 (21.37)	S	Italia	41.17	Csa
Caserta, [86]	2007–2008	11–13	575	288 (50.08)	287 (49.92)	82 (14.26)	179 (31.13)	53 (18.4)	90 (31.25)	29 (10.1)	89 (31)	S	Italia	41.17	Csa
Celi, [87]	1993–2001	6–14	34080	17371 (50.97)	16709 (49.03)	2417 (7.09)	7491 (21.98)	1235 (7.1)	3970 (22.85)	1182 (7.07)	3521 (21.07)	S	Italia	41.17	Csa
Lazzeri, [88]	2010	11–13	41389	20844 (50.36)	20545 (49.64)	1538 (3.71)	7877 (19.03)	1055 (5.06)	4737 (22.72)	483 (2.35)	3140 (15.28)	S	Italia	41.17	Csa
Toselli, [89]	2007–2008	6–11	1432	728 (50.83)	704 (49.17)	115 (8.03)	304 (21.22)	50 (6.86)	151 (20.7)	65 (9.23)	153 (21.74)	S	Italia	41.17	Cfb
Velluzzi, [90]	2003	12–14	3946	2011 (51)	1935 (49)	147 (3.7)	593 (15)	92 (4.57)	310 (15.4)	55 (2.84)	283 (14.6)	S	Italia	41.17	Csa
Wijnhoven, [42]	2007–2010	8–9	41672	21477 (51.5)	20195 (48.5)	4435 (10.64)	9489 (22.77)	2367 (11.02)	4831 (22.5)	2068 (10.09)	4658 (23.06)	S	Italia	41.17	Csa

First author, reference number	Survey period	Subject characteristic		N by gender		Results						Region	Country	Latitude	Climate zone
						Together		Boys		Girls					
		Age	N	Boys (%)	Girls (%)	Ob (%)	Ow (%)	Ob (%)	Ow (%)	Ob (%)	Ow (%)				
Karklina, [91]	2007 – 2009	9–10	504	266 (52.77)	238 (47.23)	32 (6.35)	85 (16.86)	16 (6.01)	35 (13.15)	16 (6.72)	50 (21)	N	Latvia	56.52	Dfb
Wijnhoven, [42]	2007–2010	7	2838	1381 (48.7)	1457 (51.3)	149 (5.25)	330 (11.63)	72 (5.2)	148 (10.7)	77 (5.3)	182 (12.5)	N	Latvia	56.52	Dfb
Smetanina, [92]	2000; 2010	7–13	2937	1413 (48.1)	1524 (51.9)	156 (5.31)	398 (13.55)	83 (5.87)	176 (12.45)	73 (4.8)	222 (14.56)	N	Litvania	55.1	Dfb
Tutkuviene, [93]	2000–2002	7–13	2859	1196 (41.83)	1663(58.17)	30 (1.04)	175 (6.12)	17 (1.36)	88 (7.33)	13 (0.8)	87 (5.24)	N	Litvania	55.1	Dfb
Wijnhoven, [42]	2007–2010	7–9	6721	3307 (49.2)	3414 (50.8)	326 (4.85)	822 (12.23)	160 (4.83)	398 (12.03)	166 (4.86)	424 (12.41)	N	Litvania	51.09	Dfb
Decelis, [94]	2012	10–11	811	412 (50.8)	399 (49.2)	115 (14.2)	165 (20.4)	61 (14.8)	100 (24.2)	54 (13.6)	65 (16.4)	S	Malta	35.56	Csa
Farrugia Sant Angelo, [95*]	2008; 2010	8.68	3089	1558 (50.44)	1531 (49.56)	583 (18.9)	768 (24.9)	317 (20.3)	430 (27.6)	266 (17.4)	338 (22.1)	S	Malta	35.56	Csa
Aurica, [96]	2019	7–10	268	136 (50.74)	132 (49.26)	22 (8.2)	59 (22)	15 (11)	31 (21.8)	7 (5.3)	28 (21.2)	E	Moldavia	46.58	Dfb
Jakšić, [97]	2012–2013	7–12	1134	565 (49.82)	568 (50.18)	68 (6)	240 (21.16)	43 (7.61)	133 (23.53)	25 (4.4)	107 (18.83)	SE	Montenegro	42.42	Cfb
Martinovic, [98]	2012–2013	7–13	4097	2076 (50.67)	2021 (49.33)	216 (5.27)	721 (17.6)	146 (7.0)	405 (19.5)	70 (3.46)	316 (15.63)	SE	Montenegro	42.42	Csa
Trap, [36]	2013–2015	11.1±2.3	1685	893 (53)	792 (47)	157 (9.31)	369 (21.9)	96 (10.75)	219 (24.52)	61 (7.7)	150 (18.93)	SE	Montenegro	42.42	Csa
Brug, [40]	2010	10–12	901	453 (50.27)	448 (49.73)	31 (3.44)	145 (16.09)	20 (4.5)	76 (16.8)	11 (2.5)	69 (15.4)	W	Nederlands	52.12	Cfb
de Wilde, [99]	1999–2007	7–10	3283	1636 (49.83)	1647 (50.17)	234 (7.12)	636 (19.37)	125 (7.64)	312 (19.07)	109 (6.61)	324 (19.67)	W	Nederlands	52.12	Cfb
Yngve, [15]	2003	11.7±0.4	694	322 (46.39)	372 (53.61)	5 (0.72)	52 (7.49)	4 (1.24)	31 (9.62)	1 (0.26)	21 (5.64)	W	Nederlands	52.12	Cfb
Trap, [36]	2013–2015	10.6±2.4	2902	1801 (50)	1801 (50)	216 (7.44)	589 (20.29)	128 (7.1)	279 (15.49)	88 (4.88)	310 (17.21)	SE	North Macedonia	41.36	Cfb
Wijnhoven [42]	2007–2010	7	2744	1429 (52.07)	1315(48.93)	282(10.27)	394 (14.35)	156 (10.9)	219 (15.3)	126 (9.6)	175 (13.3)	SE	North Macedonia	41.36	Cfb
Brug, [40]	2010	10–12	978	470 (48.06)	508 (51.94)	14 (1.43)	141 (14.41)	2 (0.4)	71 (15.1)	12 (2.4)	70 (13.8)	N	Norway	64.34	Dfc
Juliusson, [100]	2003–2006	6–14	3137	1591 (50.7)	1546 (49.3)	98 (3.12)	432 (13.77)	51 (3.2)	229 (14.4)	47 (3.04)	203 (13.13)	N	Norway	64.34	Cfb
Wijnhoven, [42]	2007–2010	8	2621	1335 (50.93)	1286 (49.07)	110 (4.2)	414 (15.81)	65 (4.9)	183 (13.7)	45 (3.5)	231 (18.0)	N	Norway	64.34	Dfc
Yngve, [15]	2003	11.3±0.3	693	340 (49.06)	353 (50.94)	9 (1.30)	81 (11.68)	6 (1.76)	44 (12.94)	3 (0.85)	37 (10.48)	N	Norway	64.34	Dfc
Kulaga, [101]	2007–2009	7–14	10391	5154 (49.6)	5237 (50.4)	421 (4.05)	1521 (14.63)	259 (5.02)	789 (15.3)	162 (3.09)	732 (14)	C	Poland	51.55	Dfb
Malecka–Tendera, [102]	2001	7–9	2916	1471 (50.44)	1445 (49.46)	106 (3.63)	345 (11.83)	53 (3.6)	169 (11.48)	53 (3.7)	176 (12.17)	C	Poland	51.55	Dfb
Poplawska, [103]	2000	6–14	1997	1005 (50.32)	992 (49.68)	60 (3)	179 (8.96)	29 (2.88)	76 (7.56)	31 (3.12)	103 (10.38)	C	Poland	51.55	Dfb
Antunes, [104]	2005–2006	6–14	1276	619 (48.52)	657 (51.48)	79 (6.19)	245 (19.2)	46 (7.47)	109 (17.69)	33 (5.02)	136 (20.7)	S	Portugal	39.33	Csa
Bingham, [105]	2009–2010	6–10	11377	5560 (48.9)	5817 (51.1)	1031 (9.06)	2436 (21.41)	481 (8.65)	1099 (19.76)	550 (9.45)	1337 (23)	S	Portugal	39.33	Csa
Ferreira, [106]	2004	6–10	1125	581 (51.6)	544 (48.4)	142 (12.6)	259 (23)	64 (11)	144 (24.8)	78 (14.3)	115 (21.2)	S	Portugal	39.33	Csa
Marques–Vidal, [107]	2000–2002	10–14	2378	1182 (49.7)	1196 (50.3)	158 (6.64)	516 (21.7)	82 (6.93)	229 (19.37)	76 (6.35)	287 (24)	S	Portugal	39.33	Csa
Mota, [108]	2006	8.3±1.1	255	127 (49.8)	128 (50.2)	33 (12.94)	76 (29.8)	17 (13.38)	39 (30.7)	16 (12.5)	37 (28.9)	S	Portugal	39.33	Csa
Padez, [109]	2002–2003	7–9	4511	2234 (49.52)	2274 (50.48)	510 (11.3)	914 (20.3)	230 (10.29)	428 (19.15)	280 (12.31)	486 (21.37)	S	Portugal	39.33	Csa
Pereira, [110]	2010	6–10	3699	1886 (51)	1813 (49)	472 (12.76)	746 (20.16)	232 (12.3)	332 (17.6)	240 (13.2)	414 (22.8)	S	Portugal	39.33	Csa
Rito, [111]	2007–2008	6–8	3763	1892 (50.27)	1871 (49.73)	335 (8.9)	721 (19.16)	183 (9.7)	384 (20.3)	152 (8.1)	337 (18)	S	Portugal	39.33	Csa
Seabra, [112]	2010	8–10	682	358 (52.5)	324 (47.5)	84 (12.31)	194 (28.44)	41 (11.5)	107 (29.9)	43 (13.3)	87 (26.9)	S	Portugal	39.33	Csa
Wijnhoven, [42]	2007–2010	7	1813	910 (50.2)	903 (49.8)	159 (8.76)	323 (17.81)	72 (7.9)	134 (14.8)	87 (9.6)	189 (20.9)	S	Portugal	39.33	Csa
Yngve, [15]	2003	11.5±0.4	1167	552 (47.3)	645 (52.7)	48 (4.11)	212 (18.16)	34 (6.16)	112 (20.29)	14 (2.17)	100 (15.5)	S	Portugal	39.33	Csa

First author, reference number	Survey period	Subject characteristic		N by gender		Results						Region	Country	Latitude	Climate zone
						Together		Boys		Girls					
		Age	N	Boys (%)	Girls (%)	Ob (%)	Ow (%)	Ob (%)	Ow (%)	Ob (%)	Ow (%)				
Radu, [113]	2004	11–14	1625	789 (48.55)	836 (51.45)	83 (5.1)	162 (9.96)	39 (4.94)	80 (10.13)	44 (5.26)	82 (9.8)	C	Romania	45.56	Dfb
Valean, [114]	2009	6–14	5256	2639 (50.2)	2617 (49.8)	555 (10.55)	811 (15.43)	344 (13.03)	428 (16.21)	211 (8.06)	383 (14.63)	C	Romania	45.56	Dfb
Klimatskaya, [38]	2010	11	1000	500 (50)	500 (50)	26 (2.6)	111 (11.1)	14 (2.8)	56 (11.2)	12 (2.4)	55 (11)	E	Russia	47.09	Dfb
Tudor–Locke, [115]	1992–1993; 1994–2002	7–13	6780	3507 (51.72)	3273 (48.28)	242 (3.5)	488 (7.2)	144 (4.1)	298 (8.5)	98 (3)	190 (5.8)	E	Russia	55.45	Dfb
Djordjic, [116]	2015	7.7±0.6	4851	2465 (50.9)	2386 (49.1)	335 (6.9)	786 (16.2)	176 (7.12)	389 (15.77)	168 (7.05)	389 (16.31)	S	Serbia	44.12	Cfb
Rakic, [117]	2001–2004; 2011–2014	7–14	3694	1674 (45.3)	2020 (54.7)	196 (5.3)	698 (18.9)	101 (6.03)	358 (21.38)	95 (4.7)	340 (16.83)	S	Serbia	44.12	Dfb–Cfb
Tisha, [118]	2015	7–8	2795	1402 (50.1)	1393 (49.9)	237 (8.47)	370 (13.23)	124 (8.8)	194 (13.8)	113 (8.1)	176 (12.6)	C	Slovakia	48.4	Dfb
Brug, [40]	2010	10–12	1148	559 (48.7)	589 (51.3)	65 (5.66)	309 (26.91)	42 (7.5)	177 (31.7)	23 (3.9)	132 (22.5)	S	Slovenia	46.08	Cfb
Kovac, [119]	1991–2006	7–12	100545	51827 (51.5)		6035 (6)	18306 (18.2)	3082 (5.94)	9473 (18.27)			S	Slovenia	46.08	Cfb
Leskosek, [120]	1991–2006	7–12	100545		48718 (48.5)	6035 (6)	18306 (18.2)			2953 (6.06)	8833 (18.13)	S	Slovenia	46.08	Cfb
Wijnhoven, [42]	2007–2010	69–	15938	8202 (51.5)	7736 (48.5)	1169 (7.33)	2409 (15.11)	595 (7.25)	1242 (15.14)	574 (7.41)	1167 (15.08)	S	Slovenia	46.08	Cfb
Ara, [121]	2007	9.7±0.1	1068	558 (52.24)	510 (47.76)	68 (6.3)	332 (31.08)	28 (5)	165 (29.6)	40 (8)	167 (32.8)	S	Spain	40.12	Csa
Brug, [40]	2010	10–12	1008	485 (48.12)	523 (51.88)	30 (2.97)	249 (24.7)	14 (2.9)	125 (25.8)	16 (3.1)	124 (23.8)	S	Spain	40.12	Csa
Gulías–González, [122]	2010	6–14	2206	1228 (55.67)	978 (44.33)	197 (8.93)	512 (23.2)	112 (9.12)	259 (21.1)	85 (8.69)	253 (25.86)	S	Spain	40.12	Csa
Laguna, [123]	2010		439	206 (43.9)	233 (56.1)	34 (7.8)	108 (24.7)	13 (6.5)	52 (25.5)	21 (9)	56 (24)	S	Spain	40.12	Csa
Martin, [124*]	1992; 2004–2006	9.2±0.5	1312	672 (51.2)	640 (48.8)	110 (8.4)	354 (27)	65 (9.6)	181 (26.93)	45 (7.1)	173 (27.03)	S	Spain	40.12	Csa
Martınez–Vizcaıno, [125]	2004	6–11	1166	609 (52.2)	557 (47.8)	108 (9.26)	256 (21.95)	66 (10.1)	133 (21.9)	42 (7.6)	123 (22.1)	S	Spain	40.12	Csa
Pérez–Farinós, [126]	2010–2011	9–10	7659	3931 (51.33)	3728 (48.67)	843 (11)	1853 (24,19)	427 (10.86)	935 (23.78)	416 (11.15)	918 (24.62)	S	Spain	40.12	Csa
Serra–Majem, [127]	1998–2000	6–9	990	492 (49.7)	498 (50.3)	70 (7.07)	300 (30.3)	44 (8.94)	172 (35)	26 (5.14)	128 (25.8)	S	Spain	40.12	Csa
Wijnhoven, [42]	2007–2010	6–13	7656	3839 (50.14)	3817 (49.86)	744 (9.71)	1851 (24.17)	366 (9.53)	926 (24.12)	378 (9,9)	925 (24.23)	S	Spain	40.12	Csa
Yngve, [15]	2003	6–9	761	389 (51.11)	372 (48.89)	18 (2.36)	119 (15.63)	13 (3.41)	66 (16.96)	5 (1.34)	53 (14.24)	S	Spain	40.12	Csa
Garmy, [128]	2008–2009	11.4±0.4	1891	963 (50.93)	928 (49.07)	64 (3.38)	249 (13.16)	33 (3.42)	117 (12.14)	31 (3.34)	132 (14.22)	N	Sweden	62.11	Dfb
Marild, [129]	2000–2001	7–14	4730	2405 (50.84)	2325 (49.16)	138 (2.91)	863 (18.24)	68 (2.82)	409 (17.0)	70 (3.01)	454 (19.52)	N	Sweden	62.11	Dfb
Moraeus, [130]	2008–2010; 2013	9–11	1328	643 (48.4)	685 (51.6)	41 (3.1)	250 (18.8)	19 (2.9)	105 (16.4)	22 (3.3)	145 (21.3)	N	Sweden	62.11	Dfb
Petersen, [131]	2001	8.4±0.6	1115	580 (52)	535 (48)	53 (4.75)	205 (18.38)	16 (2.75)	101 (17.41)	37 (6.91)	104 (19.43)	N	Sweden	62.11	Dfc
Sjöberg, [132]	2008	6–13	4538	2371 (52.2)	2167 (47.8)	134 (3.0)	754 (16.6)	59 (2.5)	381 (16.1)	75 (3.5)	373 (17.2)	N	Sweden	62.11	Dfb
Sundblom, [133]	1999; 2003	8.4±0.6	2183	1132 (51.85)	1051 (48.15)	73 (3.34)	434 (19.87)	43 (3.8)	232 (20.49)	30 (2.85)	202 (19.21)	N	Sweden	62.11	Dfb
Yngve, [15]	2003	10.5±0.40	925	441 (47.67)	484 (52.33)	14 (1.51)	97 (10.42)	11 (2.49)	58 (13.15)	3 (0.61)	39 (8.05)	N	Sweden	62.11	Dfb
Chiolero, [134]	2005–2006	11.4±0.5	5207	2621 (50.3)	2586 (49.7)	91 (1.74)	626 (12.02)	47 (1.79)	349 (13.31)	44 (1.7)	277 (10.71)	W	Switzerland	46.48	Dfb
Jeannot, [135]	2011–2012	12.3±0.5	8544	4232 (49.53)	4312 (50.47)	272 (3.18)	1178 (13.78)	136 (3.2)	572 (13.5)	136 (3.15)	606 (14.04)	W	Switzerland	46.48	Cfb–Dfb

First author, reference number	Survey period	Subject characteristic		N by gender		Results						Region	Country	Latitude	Climate zone
						Together		Boys		Girls					
		Age	N	Boys (%)	Girls (%)	Ob (%)	Ow (%)	Ob (%)	Ow (%)	Ob (%)	Ow (%)				
Zimmermann, [136]	2000	6–14	595	297 (49.9)	298 (50.1)	58 (9.74)	143 (24.03)	27 (9.2)	69 (23.1)	31 (10.4)	74 (24.7)	W	Switzerland	46.48	Dfb
Zimmermann, [137]	2004	6–12	2431	1196 (49.2)	1235 (50.8)	92 (3.78)	435 (17.89)	46 (3.84)	199 (16.63)	46 (3.72)	236 (19.11)	W	Switzerland	46.48	Dfb
Agirbasli, [138]	1989; 2008	9.8±1.8	1552	761 (49.03)	791 (50.97)	77 (4.96)	241 (15.52)	34 (4.5)	114 (15)	33 (4.2)	127 (16)	SE	Turkey	41	Csa
Dundar, [139]	2012	12.8±0.9	2477	1271 (51.3)	1206 (48.7)	254 (10.25)	554 (22.36)	138 (10.9)	354 (27.9)	116 (9.6)	200 (16.6)	SE	Turkey	41	Csa
Manios, [140]	2001	12–13	510	257 (50.4)	253 (49.6)	8 (1.6)	54 (10.6)	4 (1.6)	25 (9.7)	4 (1.6)	25 (11.5)	SE	Turkey	41	Csa
Oner, [141]	2001	12–14	593	300 (50.6)	293 (49.4)	10 (1.68)	69 (11.63)	5 (1.66)	35 (11.66)	5 (1.7)	34 (11.6)	SE	Turkey	41	Csa
Ozer, [142]	2005	6–14	1101	542 (49.23)	559 (50.77)	45 (4.08)	242 (22)	25 (4.61)	113 (20.84)	20 (3.57)	129 (23.07)	SE	Turkey	41	Csa
Pirincci, [143]	2007	6–12	3642	1860 (50.08)	1782 (48.92)	60 (1.6)	481 (13.2)	38 (2)	259 (13.9)	22 (1.2)	222 (12.5)	SE	Turkey	41	Csa
Simsek, [144]	2005–2006	6–14	4535	2198 (51.53)	2337 (48.47)	256 (5.64)	951 (21)	60 (2.75)	453 (20.61)	196 (5.38)	498 (21.31)	SE	Turkey	41	Csa
Sur, [145]	2001–2002	12–13	1044	516 (49.2)	528 (50.8)	21 (2.0)	124 (11.9)	9 (1.7)	68 (13.2)	12 (2.3)	56 (10.6)	SE	Turkey	41	Csa
Høyer, [77*]	2010–2012	6–9	488	260 (53.27)	228 (46.73)	29 (5.94)	66 (13.52)	18 (6.92)	36 (13.84)	11 (4.82)	30 (13.15)	E	Ukraine	48.22	Dfb
Dereń, [146]	2018	6–12	8175	3997 (48.9)	4178 (51.1)	259 (3.17)	1032 (12.62)	142 (3.4)	564 (13.5)	117 (2.8)	468 (11.2)	E	Ukraine	48.22	Dfb
Dereń, [147]	2018–2019	7–14	15645	7602 (48.59)	8043 (51.41)	399 (2.55)	2014 (12.89)	174 (2.28)	882 (11.61)	225 (2.8)	1132(14.07)	E	Ukraine	48.22	Dfb

*Legends: Ob–Obesity; Ow–Overweight; N–Number; B–Boys; G–Girls; S–South; SE–Southeast; C–Central; E–East; W–West; N–North; *–Cross sectional analysis at retrospectional or prospective cohort study (one time point).*

Note: Greenland has Danish and Inuit population and is an autonomous territory within the Kingdom of Denmark and is the only Danish territory associated to the EU (eeas.europa.eu), also see in Appendix in (13), For Koppen–Geiger markings see in (30).

Table S3. Quality assessment of prevalence studies following the the Joanna Briggs Institute tool

First author and reference number	Was the sample representative of the target population?	Were study participants recruited in an appropriate way?	Was the sample size adequate?	Were the study subjects and the setting described in detail?	Was the data analysis conducted with sufficient coverage of the identified sample?	Were objective, standard criteria used for the measurement of the condition?	Was the condition measured reliably?	Was there appropriate statistical analysis?	Are all important confounding factors/subgroups/differences identified and accounted for?	Were subpopulations identified using objective criteria?
Hyska, 35										
Trap, 36										
Mayer, 37										
Yngve, 15										
Klimatskaya, 38										
Marfina, 39										
Brug, 40										
Roelants, 41										
Wijnhoven, 42										
Mladenova, 43										
Mladenova, 44										
Milanovic, 45										
Milanovic, 46										
Savva, 47										
Savva, 48										
Kunešová, 49										
Kunešová, 50										
Matthiessen, 51										
Basterfield, 52										
Dummer, 53										
Harding, 54										
Hughes, 55										
Stamatakis, 56										
Steele, 57										
Eloranta, 58										
Stigman, 59										
Rolland–Cachera, 60										
Salanava, 61										
Klein–Platat, 62										
Heude, 63										
Thibault, 64										
Bluher, 65										
Kromeyer–Hauschild, 66										
Nagel, 67										
Will, 68										
Angelopoulos, 69										
Grigorakis, 70										
Hassapidou, 71										
Manios, 72										

First author and reference number	Was the sample representative of the target population?	Were study participants recruited in an appropriate way?	Was the sample size adequate?	Were the study subjects and the setting described in detail?	Was the data analysis conducted with sufficient coverage of the identified sample?	Were objective, standard criteria used for the measurement of the condition?	Was the condition measured reliably?	Was there appropriate statistical analysis?	Are all important confounding factors/subgroups/differences identified and accounted for?	Were subpopulations identified using objective criteria?
Manios, 73	Green	Green	Green	Yellow	Green	Green	Yellow	Green	Green	Green
Papadimitriou, 74	Green	Green	Green	Green	Green	Green	Green	Green	Yellow	Green
Tambalis, 75	Green	Green	Green	Yellow	Yellow	Green	Green	Green	Yellow	Yellow
Tokmakidis, 76	Green	Green	Green	Yellow	Green	Green	Yellow	Green	Green	Yellow
Høyer, 77	Green	Green	Green	Green	Green	Yellow	Yellow	Green	Yellow	Yellow
Rex, 78	Green	Green	Green	Yellow	Green	Green	Green	Green	Yellow	Yellow
Barron, 79	Red	Yellow	Green	Green	Green	Green	Yellow	Green	Green	Green
O’Neill, 80	Red	Green	Green	Yellow	Yellow	Green	Green	Green	Yellow	Green
Watkins, 81	Red	Green	Green	Yellow	Green	Green	Yellow	Green	Yellow	Green
Whelton, 82	Green	Green	Green	Green	Green	Green	Green	Green	Green	Green
Albertini, 83	Green	Yellow	Green	Green	Green	Yellow	Yellow	Yellow	Green	Green
Baratta, 84	Red	Yellow	Green	Yellow	Green	Green	Green	Green	Green	Green
Bertoncello, 85	Green	Green	Green	Green	Green	Yellow	Yellow	Green	Green	Green
Caserta, 86	Green	Green	Green	Green	Yellow	Yellow	Yellow	Green	Yellow	Yellow
Celi, 87	Red	Green	Green	Yellow	Green	Yellow	Yellow	Green	Green	Green
Lazzeri, 88	Red	Green	Green	Green	Green	Green	Red	Green	Green	Green
Toselli, 89	Green	Green	Green	Yellow	Green	Green	Green	Green	Yellow	Yellow
Velluzzi, 90	Red	Green	Green	Green	Green	Green	Green	Green	Green	Green
Karklina, 91	Green	Green	Green	Green	Green	Green	Green	Yellow	Green	Green
Smetanina, 92	Red	Green	Green	Yellow	Green	Green	Green	Green	Yellow	Green
Tutkuviene, 93	Red	Green	Green	Yellow	Yellow	Yellow	Green	Green	Yellow	Green
Decelis, 94	Green	Yellow	Green	Yellow	Green	Green	Green	Green	Green	Green
Sant’Angelo, 95	Green	Green	Green	Yellow	Green	Green	Green	Green	Yellow	Green
Aurica, 96	Green	Green	Green	Green	Green	Green	Green	Green	Yellow	Yellow
Jakšić, 97	Green	Green	Green	Green	Green	Green	Green	Green	Green	Green
Martinovic, 98	Green	Green	Green	Green	Green	Green	Green	Green	Green	Green
de Wilde, 99	Red	Green	Green	Yellow	Green	Yellow	Green	Green	Green	Green
Juliusson, 100	Red	Green	Green	Green	Green	Green	Green	Green	Green	Green
Kulaga, 101	Red	Green	Green	Yellow	Green	Green	Green	Green	Yellow	Yellow
Malecka–Tendera, 102	Green	Green	Green	Green	Green	Green	Green	Green	Green	Green
Poplawska, 103	Red	Yellow	Green	Yellow	Green	Yellow	Yellow	Green	Yellow	Yellow
Antunes, 104	Green	Green	Green	Green	Green	Green	Green	Green	Green	Green
Bingham, 105	Red	Green	Green	Green	Green	Green	Green	Green	Green	Green
Ferreira, 106	Green	Green	Green	Yellow	Green	Yellow	Yellow	Green	Green	Green
Marques–Vidal, 107	Red	Yellow	Green	Yellow	Green	Green	Green	Green	Blue	Blue
Mota, 108	Green	Green	Green	Yellow	Green	Green	Green	Green	Yellow	Yellow
Padez, 109	Green	Green	Green	Green	Green	Green	Green	Green	Green	Green
Pereira, 110	Green	Green	Green	Yellow	Green	Green	Green	Green	Green	Green
Rito, 111	Green	Green	Green	Green	Green	Green	Green	Green	Green	Green
Seabra, 112	Green	Green	Green	Yellow	Green	Green	Green	Green	Green	Green

First author and reference number	Was the sample representative of the target population?	Were study participants recruited in an appropriate way?	Was the sample size adequate?	Were the study subjects and the setting described in detail?	Was the data analysis conducted with sufficient coverage of the identified sample?	Were objective, standard criteria used for the measurement of the condition?	Was the condition measured reliably?	Was there appropriate statistical analysis?	Are all important confounding factors/subgroups/differences identified and accounted for?	Were subpopulations identified using objective criteria?
Radu, 113	No	Unclear	Yes	Unclear	Yes	Unclear	Unclear	Yes	Unclear	Unclear
Valean, 114	No	Unclear	Yes	Unclear	Yes	Unclear	Unclear	Yes	Unclear	Unclear
Tudor–Locke, 115	Yes	Unclear	Yes	Unclear	Yes	Unclear	Unclear	Yes	Unclear	Unclear
Djordjic, 116	Yes	Yes	Yes	Yes	Yes	Yes	Yes	Unclear	Unclear	Unclear
Rakic, 117	No	Yes	Yes	Yes	Yes	Yes	Yes	Yes	Yes	Yes
Tisha, 118	Yes	Yes	Yes	Yes	Yes	Yes	Yes	Yes	Yes	Yes
Kovac, 119	No	Unclear	Yes	Unclear	Yes	Unclear	Unclear	Unclear	Unclear	Yes
Leskosek, 120	No	Unclear	Yes	Unclear	Yes	Unclear	Unclear	Unclear	Unclear	Yes
Ara, 121	Yes	Yes	Yes	Unclear	Yes	Yes	Yes	Yes	Yes	Yes
Gulías–González, 122	No	Yes	Yes	Yes	Yes	Yes	Unclear	Yes	Yes	Yes
Laguna, 123	Yes	Yes	Yes	Yes	Yes	Yes	Yes	Yes	Unclear	Yes
Martin, 124	No	Yes	Yes	Unclear	Yes	Yes	Yes	Yes	Not applicable	Unclear
Martınez–Vizcaıno, 125	Yes	Yes	Yes	Unclear	Yes	Yes	Yes	Yes	Yes	Yes
Pérez–Farinós, 126	Yes	Yes	Yes	Yes	Yes	Yes	Yes	Yes	Yes	Yes
Serra–Majem, 127	No	Yes	Yes	Yes	Yes	Unclear	Unclear	Unclear	Unclear	Unclear
Garmy, 128	No	Yes	Yes	Yes	Yes	Unclear	Unclear	Yes	Yes	Yes
Marild, 129	Yes	Unclear	Yes	Yes	Yes	Yes	Yes	Yes	Yes	Yes
Moraeus, 130	Yes	Yes	Yes	Unclear	Yes	Yes	Yes	Yes	Yes	Yes
Petersen, 131	Yes	Yes	Yes	Yes	Yes	Yes	Yes	Yes	Yes	Yes
Sjöberg, 132	Yes	Yes	Yes	Unclear	Yes	Yes	Yes	Yes	Yes	Yes
Sundblom, 133	Yes	Unclear	Yes	Unclear	Yes	Unclear	Unclear	Unclear	Unclear	Yes
Chiolero, 134	Yes	Yes	Yes	Unclear	Yes	Yes	Yes	Unclear	Unclear	Unclear
Jeannot, 135	No	Unclear	Yes	Unclear	Yes	Yes	Unclear	Yes	Yes	Yes
Zimmermann, 136	Yes	Yes	Yes	Unclear	Yes	Yes	Yes	Unclear	Unclear	Unclear
Zimmermann, 137	Yes	Yes	Yes	Yes	Yes	Yes	Yes	Yes	Yes	Yes
Agirbasli, 138	No	Yes	Yes	Unclear	Yes	Unclear	Unclear	Yes	Yes	Yes
Dundar, 139	Yes	Yes	Yes	Yes	Yes	Yes	Yes	Yes	Unclear	Yes
Manios, 140	Yes	Yes	Yes	Yes	Yes	Yes	Yes	Yes	Yes	Yes
Oner, 141	No	Yes	Yes	Unclear	Yes	Yes	Yes	Unclear	Yes	Yes
Ozer, 142	No	Yes	Yes	Yes	Yes	Yes	Yes	Yes	Unclear	Yes
Pirincci, 143	Yes	Yes	Yes	Unclear	Yes	Yes	Yes	Yes	Yes	Yes
Simsek, 144	No	Yes	Yes	Unclear	Yes	Yes	Yes	Yes	Yes	Yes
Sur, 145	Yes	Yes	Yes	Yes	Yes	Yes	Yes	Yes	Yes	Yes
Dereń, 146	No	Yes	Yes	Yes	Yes	Yes	Yes	Yes	Unclear	Yes
Deren, 147	No	Yes	Yes	Yes	Yes	Yes	Yes	Yes	Unclear	Yes

Yes – (green) No – (red) Unclear – (yellow) Not applicable – (blue)

Table S4. Number of Koppen-Geiger climate zones per country

Number per country		
**One**	**Two**	**Three or more**
BLR, ENG, HUN, IRL, LVA, LTU, MLT, and POL	BEL, CYP, CZE, DNK, FIN, DEU, GRL, MDA, and SVK	ALB, AUS, BGR, HRV, FRA, GRC, ISL, ITA, MNE, NLD, MKD, NOR, PRT, ROU, RUS, SRB, SVN, ESP, SWE, CHE, TUR, and UKR

Note: From all the studies we collected and represented subject data of place of residence in view of precise latitude. We found and determined the climate zone for that country and in case that country had different subject places of residence and different climate zones (depending on different studies), we would make a decision based on the ratio of the number of subjects. This was the case only in BGR, HRV, GRl, ISL, MNE, MKD, SRB, and SVN; The three-letter country abbreviations can be found in Table S1.

Table S5. Prevalence from 2000 to 2020 and overall (pool estimate (95% confidence interval) of obesity and overweight in European children aged 6–14 years by quality of included studies

Quality	2000–2009			2010–2020			2000–2020		
	**N**	**95% CI**	**I^2**	**N**	**95% CI**	**I^2**	**N**	**95% CI**	**I^2**
	**Boys**								
Low	54	17.58 (16.28–18.96)	95.88	28	19.43 (17.52–21.49)	99.25	82	18.04 (16.90–19.23)	98.68
		5.18 (4.51–5.94)	95.69		7.54 (6.57–8.64)	98.71		6.01 (5.45–6.62)	97.95
Moderate	21	17.81 (15.64–20.21)	98.65	4	18.26 (13.21–24.70)	98.6	25	17.60 (15.38–20.08)	98.52
		5.30 (4.21–6.65)	98.39		7.47 (4.90–11.20)	98.18		5.54 (4.53–6.75)	98.23
High	6	12.74 (9.61–16.71)	96.61	1	13.73 (7.56–23.67)	83.73	7	12.98 (9.84–16.93)	95.51
		5.00 (3.12–7.92)	98.34		4.13 (1.90–8.76)	81.13		4.77 (3.23–6.99)	97.62
All studies	81	17.32 (16.24–18.45)	97.57	33	19.11 (17.35–21.01)	99.26	114	17.68 (16.69–18.71)	98.66
		5.20 (4.64–5.83)	97.39		7.40 (6.51–8.41)	98.77		5.76 (5.11–6.48)	98.07
	**Girls**								
Low	54	17.68 (16.35–19.10)	94.66	28	18.90 (17.29–20.63)	98.95	82	17.92 (16.89–19.01)	98.17
		4.89 (4.26–5.60)	93.98		6.57 (5.77–7.46)	98.32		5.51 (5.02–6.06)	97.34
Moderate	21	16.82 (14.71–19.17)	98.91	4	17.79 (13.41–23.21)	98.58	25	16.70 (14.73–18.86)	98.75
		4.35 (3.47–5.46)	98.49		5.59 (3.70–8.35)	98.12		4.42 (3.62–5.36)	98.32
High	6	12.15 (9.09–16.07)	96.96	1	13.04 (7.76–21.11)	75.54	7	12.38 (9.61–15.82)	95.95
		4.42 (2.78–6.98)	93.01		2.87 (1.33–6.05)	0		3.98 (2.72–5.79)	91.92
All studies	81	17.10 (16.01–18.25)	97.51	33	18.59 (17.09–20.18)	99.02	114	17.39 (16.50–18.31)	98.33
		4.72 (4.20–5.29)	96.86		6.33 (5.60–7.14)	98.47		4.78 (3.85–5.93)	97.61

Legends: N–Number of studies.

Note: In each quality column top row show overweight and botom row obesity results.

Table S6. Prevalence by European regions through 2000–2020 and overall (pool estimate (95% confidence interval) of obesity and overweight in European children aged 6–14 years of included studies

Regions (Sn)	2000–2009			2010–2020			2000–2020			Trends
	N/Cn	95% CI	I^2	N/Cn	95% CI	I^2	N/Cn	95% CI	I^2	
	**Boys**									
Central (33377)	15/6	14.54 (12.75–16.52)	97.36	6/5	15.58 (12.93–18.64)	93.66	21/8	14.84 (13.33–16.48)	96.81	1.04
		4.40 (3.51–5.50)	96.67		5.95 (4.33–8.11)	93.81		4.84 (4.06–5.78)	96.03	1.55
East (17188)	3/3	13.07 (9.60–17.57)	95.03	5/3	13.35 (10.77–16.43)	83.87	8/4	13.24 (11.05–15.79)	91.9	0.28
		4.59 (2.74–7.57)	90.57		3.83 (2.65–5.50)	90.72		4.09 (3.04–5.45)	90.86	–0.76
North (22089)	15/7	13.43 (11.71–15.37)	89.02	8/5	13.78 (11.65–16.23)	76.69	23/8	13.57 (12.19–15.08)	87.45	0.35
		2.83 (2.22–3.61)	58.03		4.90 (3.64–6.57)	80.36		3.49 (2.90–4.19)	83.94	2.07
South (192071)	20/4	22.21 (20.07–24.51)	97.04	19/7	21.94 (19.96–24.05)	95.91	39/7	22.08 (20.58–23.65)	96.55	–0.27
		8.03 (6.71–9.58)	96.03		9.35 (7.90–11.03)	98.21		8.66 (7.68–9.74)	97.83	1.32
Southeast (134268)	16/3	20.67 (18.35–23.20)	96.3	13/7	21.34 (19.00–23.88)	98.23	29/7	20.99 (19.29–22.80)	97.5	0.67
		6.79 (5.48–8.40)	96.77		8.47 (6.89–10.37)	95.57		7.60 (6.57–8.77)	96.78	1.68
West (100078)	19/5	16.43 (14.64–18.38)	94.74	4/3	13.70 (10.84–17.18)	94.77	23/5	15.92 (14.38–17.60)	98.43	–2.73
		4.91 (4.00–6.01)	95.95		3.69 (3.55–3.83)	0		4.77 (4.00–5.67)	97.11	–1.22
	(Sn–234796)			(Sn–264275)			(Sn–463192)			
	**Girls**									
Central (33024)	15/6	14.36 (12.68–16.21)	95.81	6/5	15.66 (12.65–19.24)	88.78	21/8	14.73 (13.24–16.26)	94.73	1.3
		3.78 (3.03–4.93)	94.44		5.55 (3.76–8.10)	93.23		4.24 (3.53–5.09)	94.24	1.77
East (17654)	3/3	9.60 (7.06–12.94)	95.79	5/3	12.85 (10.02–16.35)	83.31	8/4	11.61 (9.64–13.92)	95.9	3.25
		2.84 (1.71–4.68)	72.1		2.85 (2.58–3.15)	3.23		2.86 (2.63–3.12)	38.17	0.01
North (22332)	15/7	13.16 (11.54–14.96)	94.45	8/5	15.93 (13.21–19.10)	88.59	23/8	14.11 (12.68–15.66)	93.01	2.77
		2.68 (2.11–3.42)	84.56		4.68 (3.30–6.58)	74.15		3.36 (2.76–4.07)	85.33	2
South (186530)	20/4	21.49 (19.51–23.61)	93.93	19/7	21.04 (18.83–23.43)	97.54	39/7	21.27 (19.81–22.80)	96.38	–0.45
		6.72 (5.67–7.93)	94.8		8.38 (6.80–10.28)	98.61		7.51 (6.61–8.51)	98.08	1.66
Southeast (130362)	16/3	20.75 (18.53–23.15)	96.5	13/7	18.68 (16.25–21.39)	98.47	29/7	19.76 (18.13–21.49)	97.8	–2.07
		6.18 (5.06–7.53)	95.36		6.14 (4.73–7.95)	97.28		6.15 (5.27–7.16)	97.14	–0.04
West (96998)	19/5	17.32 (15.55–19.24)	95.64	4/3	14.99 (11.47–19.35)	92.12	23/5	16.85 (15.22–18.61)	98.11	–2.33
		5.34 (4.44–6.41)	94.35		3.55 (2.14–5.85)	75.59		4.95 (4.14–5.92)	94.85	–1.79
	(Sn–228396)			(Sn–258504)			(Sn–522779)			

No statistical significance between genders and trends

Legend: N–Number of studies; Cn–Number of countries; Sn–Subjects number. Note: In each region column top row show overweight and bottom row obesity results.

Table S7. Pooled estimate and prevalence trends of overweight and obesity in elementary school children aged 6–14 years across 39 European countries using IOTF definition criteria (15, 35–147)

Country	Boys, 95% CI, %				Girls, 95% CI, %			
	**2000–2009**	**2010–2020**	**2000–2020**	**Trend**	**2000–2009**	**2010–2020**	**2000–2020**	**Trend**
ALB	n/a	13.99 (10.88–17.82)	14.00 (10.95–17.72)	x	n/a	13.14 (9.71–17.54)	13.15 (10.25–16.73)*	x
	n/a	4.87 (2.84–8.23)	4.88 (3.15–7.48)	x	n/a	3.42 (1.54–7.43)	3.43 (2.01–5.81)*	x
**AUT**	14.84 (12.14–18.01)f	17.00 (15.64–18.45)f	16.65 (15.42–17.95)f	2.16	11.28 (9.07–13.93)f	17.18 (15.80–18.66)f	14.36 (11.04–18.47)	5.9*
	2.12 (1.21–3.69)f	6.80 (5.92–7.80)f	4.48 (2.73–7.25)	4.68*	2.44 (1.50–3.94) f	5.59 (4.78–6.53)f	3.95 (2.23–6.89)	3.15*
**BLR**	13.37 (11.02–16.12)f	16.66 (13.64–20.20)f	14.91 (11.32–19.38)	3.29	9.02 (7.22–11.21)f*	15.33 (12.44–18.76)f	11.81 (8.85–15.61)	6.31*
	2.18 (1.32–3.58)f	5.82 (4.08–8.25)f	3.76 (2.23–6.29)	3.64*	1.63 (0.94–2.79)f	3.39 (2.12–5.38)f	2.39 (1.26–4.46)	1.76*
**BEL**	11.64 (8.23–16.22)	12.51 (9.58–16.19)*	12.18 (10.08–14.64)	0.87*	9.30 (6.67–12.80)*	12.70 (12.44–12.95)f	11.23 (9.24–13.58)*	3.4*
	2.08 (1.11–3.85)	3.67 (3.54–3.82)f	2.97 (2.06–4.26)	1.59*	2.55 (2.12–3.07)f	3.49 (1.52–7.81)*	2.97 (1.96–4.47)*	0.94*
BGR	n/a	18.77 (15.93–21.99)f	18.77 (15.93–21.99)f	x	n/a	17.45 (14.70–20.60)f	17.45 (14.70–20.60)f	x
	n/a	7.83 (5.98– 10.17)f	7.83 (5.98–10.17)f	x	n/a	4.26 (2.94–6.14)f	4.26 (2.94–6.14)f*	x
HRV	n/a	21.24 (20.03–22.51)f	21.24 (20.03–22.51)f	x	n/a	21.11 (19.90–22.38)f	21.11 (19.90–22.38)f	x
	n/a	15.03 (9.20–23.60)	15.02 (10.13–21.71)	x	n/a	9.94 (4.67–19.93)	9.93 (6.01–15.98)*	x
CYP	19.75 (14.57–26.20)	n/a	19.79 (15.62–24.74)	x	20.22 (15.42–26.06)	n/a	20.25 (15.99–25.29)	x
	8.53 (7.75–9.38)f	n/a	8.53 (7.75–9.38)f	x	6.90 (6.19–7.69)f	n/a	6.90 (6.19–7.69)f*	x
**CZE**	16.91 (11.99–23.31)	12.53 (10.18–15.34)f	15.31 (12.16–19.09)	–4.38	16.12 (11.60–21.97)	13.42 (10.98–16.31)f	15.17 (11.97–19.05)	–2.7
	7.27 (4.31–11.99)	5.02 (3.57–7.01)f	6.42 (4.33–9.41)	–2.25	5.67 (3.26–9.69)	5.85 (4.26–7.96)f	5.74 (3.58–9.06)	0.18
DNK	10.75 (8.88–12.96)f	n/a	10.75 (8.88–12.96)f	x	9.87 (6.88–13.94)	n/a	9.87 (7.11–13.54)	x
	2.34 (1.22–4.45)	n/a	2.35 (1.27–4.30)	x	1.67 (0.74–3.70)	n/a	1.63 (0.70–3.73)	x
ENG	20.46 (17.15–24.22)	n/a	20.50 (17.84–23.45)*	x	23.12 (19.83–26.77)	n/a	23.15 (20.24–26.34)	x
	7.73 (5.82–10.20)	n/a	7.76 (5.99–10.00)	x	7.87 (5.92–10.38)	n/a	7.85 (5.78–10.57)	x
FIN	10.71 (6.91–16.25)	n/a	10.80 (7.35–15.60)	x	13.98 (9.58–19.97)	n/a	13.97 (9.84–19.46)	x
	4.13 (2.58–6.55)f	n/a	4.13 (2.58–6.55)f	x	3.28 (1.43–7.31)	n/a	3.21 (1.26–7.41)	x
FRA	14.97 (12.07–18.42)	n/a	15.25 (12.83–18.04)	x	15.58 (12.85–18.78)	n/a	15.67 (13.20–18.51)	x
	3.32 (2.30–4.78)	n/a	3.38 (2.40–4.73)	x	3.48 (2.43–4.98)	n/a	3.47 (2.36–5.06)	x
DEU	15.46 (12.17–19.43)	n/a	15.88 (13.12–19.10)	x	15.79 (12.74–19.40)	n/a	15.84 (13.10–19.03)*	x
	3.36 (2.21–5.06)	n/a	3.45 (2.35–5.04)	x	2.51 (1.66–3.80)	n/a	2.51 (1.61–3.88)*	x
**GRC**	28.03 (24.03–32.41)	31.00 (26.18–36.27)	29.03 (26.32–31.90)	2.97*	28.17 (24.50–32.16)	29.23 (23.81–35.32)	28.49 (25.80–31.33)	1.05*
	12.29 (11.97–12.61)f	11.49 (7.58–17.05)	12.58 (10.41–15.13)	–0.8*	11.08 (10.77–11.39)f*	10.43 (5.65–18.47)*	10.37 (8.22–13.01)*	–0.65*
GRL	n/a	16.16 (11.90–21.59)	16.17 (11.96–21.50)	x	n/a	19.03 (15.94–22.56)f	19.03 (15.94–22.56)f	x
	n/a	7.83 (4.33–13.74)	7.87 (4.76–12.73)	x	n/a	7.95 (5.95–10.55)f	7.95 (5.95–10.55)f	x
HUN	n/a	19.13 (14.62–24.63)	19.15 (14.71–24.54)	x	n/a	18.92 (13.96–25.12)	18.93 (14.64–24.12)	x
	n/a	6.72 (5.34–8.44)f	6.72 (5.34–8.44)f	x	n/a	5.89 (2.62–12.69)	5.97 (3.40–10.25)	x

Country	Boys, 95% CI, %				Girls, 95% CI, %			
	**2000–2009**	**2010–2020**	**2000–2020**	**Trend**	**2000–2009**	**2010–2020**	**2000–2020**	**Trend**
ISL	16.19 (12.70–20.41)f<br>1.70 (0.76–3.74)f	n/a<br>n/a	16.19 (12.70–20.41)f<br>1.70 (0.76–3.74)f	x<br>x	9.55 (6.97–12.95)f<br>1.33 (0.55–3.15)f	n/a<br>n/a	9.55 (6.97–12.95)f*<br>1.33 (0.55–3.15)f	x<br>x
**IRL**	16.92 (16.00–17.89)f*<br>6.06 (5.49–6.69)f*	13.30 (11.35–15.53)f*<br>4.31 (3.22–5.74)f	15.67 (13.18–18.52)*<br>5.27 (3.84–7.18)*	–3.62*<br>–1.75*	20.08 (19.09–21.10)f<br>6.93 (4.76–9.98)	19.19 (16.82–21.80)f<br>4.77 (3.59–6.31)f	19.96 (19.04–20.91)f<br>6.41 (4.49–9.06)	–0.89<br>–2.16*
**ITA**	21.44 (18.33–24.91)<br>8.01 (6.22–10.24)	22.60 (22.21–23.00)f<br>7.52 (4.47–12.37)	21.66 (19.48–24.00)<br>7.88 (6.45–9.59)	1.16<br>–0.49	20.95 (18.19–24.02)*<br>6.47 (4.99–8.34)*	18.87 (14.27–24.53)*<br>4.98 (2.29–10.53)*	20.40 (18.31–22.66)*<br>6.10 (4.70–7.75)*	–2.08*<br>–1.49
**LVA**	13.15 (9.60–17.77)f*<br>6.02 (3.72–9.59)f	10.71 (9.19–12.46)f<br>5.21 (4.16–6.52)f	11.14 (9.71–12.75)f*<br>5.35 (4.36–6.55)f*	–2.44<br>–0.81	21.00 (16.29–26.64)f<br>6.72 (4.16–10.69)f	12.49 (10.89–14.29)f<br>5.28 (4.25–6.56)f	15.58 (11.82–20.25)<br>5.51 (4.51–6.70)f	–8.51*<br>–1.44
**LTU**	7.35 (6.01–8.98)f<br>1.42 (0.88–2.27)f	12.16 (11.26–13.12)f<br>5.32 (3.09–9.02)	10.54 (8.58–13.04)<br>3.81 (2.59–5.57)	4.81*<br>3.9*	5.23 (4.26–6.41)f*<br>0.78 (0.45–1.34)f	13.43 (9.90–17.97)<br>4.84 (4.28–5.48)f	10.25 (8.24–12.69)<br>3.03 (1.91–4.79)	8.2*<br>4.06*
MLT	n/a<br>n/a	26.92 (25.01–28.93)f<br>17.52 (10.73–27.31)	26.92 (25.01–28.93)f<br>17.59 (11.82–25.36)	x<br>x	n/a<br>n/a	19.32 (14.29–25.59)<br>15.40 (7.41–29.27)	19.44 (15.08–24.69)*<br>15.45 (9.49–24.18)*	x<br>x
MDA	22.79 (16.51–30.59)f<br>11.02 (6.76–17.49)f	n/a<br>n/a	22.79 (16.51–30.59)f<br>11.02 (6.76–17.49)f	x<br>x	21.21 (15.06–29.01)f<br>5.30 (2.50–10.70)f	n/a<br>n/a	21.21 (15.06–29.01)f<br>5.30 (2.55–10.70)f	x<br>x
MNE	n/a<br>n/a	22.34 (18.39–26.87)<br>8.34 (5.38–12.69)	22.34 (18.46–26.76)<br>8.34 (5.83–11.79)	x<br>x	n/a<br>n/a	17.73 (13.89–22.36)<br>4.92 (2.55–9.28)	17.70 (14.42–21.54)<br>4.92 (3.14–7.65)*	x<br>x
**NLD**	14.57 (10.28–20.24)<br>4.81 (2.65–8.56)	16.77 (13.61–20.50)f<br>4.42 (2.87–6.74)f	15.57 (12.41–19.35)<br>4.80 (3.10–7.38)	2.2<br>–0.39	12.95 (9.29–17.77)<br>4.74 (2.44–8.99)	15.40 (12.34–19.04)f<br>2.46 (1.36–4.38)f	14.07 (11.11–17.68)<br>3.64 (2.07–6.34)	2.45<br>–2.28*
MKD	n/a<br>n/a	15.41 (14.21–16.70)f<br>8.84 (5.23–14.55)	15.41 (14.21–16.70)f<br>8.84 (5.78–13.29)	x<br>x	n/a<br>n/a	15.19 (11.23–20.25)<br>6.88 (3.16–14.34)	15.21 (11.82–19.36)<br>6.89 (4.09–11.40)*	x<br>x
**NOR**	14.14 (12.66–15.77)f<br>3.01 (2.33–3.88)f	14.08 (12.55–15.76)f*<br>2.96 (1.45–5.93)	14.11 (13.03–15.27)f<br>2.94 (1.96–4.40)	–0.06<br>–0.05	12.67 (11.25–14.25)f<br>2.23 (1.17–4.23)	15.93 (11.65–21.40)<br>3.22 (2.49–4.15)f	13.99 (11.52–16.89)<br>2.59 (1.65–4.06)	3.26*<br>0.99
POL	11.28 (8.57–14.71)<br>3.82 (2.52–5.75)	n/a<br>n/a	11.41 (9.19–14.09)<br>3.83 (2.62–5.57)	x<br>x	12.19 (9.56–15.44)<br>3.21 (2.84–3.63)f	n/a<br>n/a	12.22 (9.87–15.04)<br>3.21 (2.84–3.63)f*	x<br>x
**PRT**	21.29 (18.07–24.91)<br>8.99 (6.92–11.60)	19.71 (16.57–23.29)*<br>9.91 (6.84–14.14)*	20.60 (18.57–22.76)*<br>9.33 (7.74–11.19)	–1.58<br>0.92	20.82 (17.93–24.03)<br>7.87 (6.00–10.25)	22.90 (22.03–23.78)f<br>11.24 (6.63–18.44)	21.66 (19.56–23.92)<br>9.01 (7.20–11.40)	2.08*<br>3.37*
ROU	13.10 (9.38–18.00)<br>8.45 (5.21–13.41)	n/a<br>n/a	13.23 (10.17–17.05)<br>8.52 (5.50–12.97)	x<br>x	12.22 (9.02–16.36)<br>6.61 (4.04–10.66)	n/a<br>n/a	12.28 (9.40–15.88)<br>6.61 (3.88–11.00)*	x<br>x
**RUS**	8.50 (7.62–9.47)f<br>4.11 (3.50–4.82)f	11.20 (8.71–14.27) f<br>2.80 (1.67–4.67)f	9.55 (7.21–12.54)<br>3.97 (3.40–4.62)f	2.7*<br>–1.31*	5.81 (5.05–6.66)f*<br>2.99 (2.46–3.64)f*	11.00 (8.54–14.05)f<br>2.40 (1.37–4.18)f	7.67 (5.73–10.19)*<br>2.92 (2.43–3.51)f*	5.19*<br>–0.59
SRB	n/a<br>n/a	18.40 (14.43–23.16)<br>6.71 (5.99–7.52)f	18.40 (14.51–23.04)<br>6.71 (5.99–7.52)f	x<br>x	n/a<br>n/a	16.54 (15.47–17.67)f<br>5.77 (2.63–12.16)	16.54 (15.47–17.67)f<br>5.78 (3.42–9.61)	x<br>x
SVK	n/a<br>n/a	13.83 (12.12–15.74)f<br>8.84 (7.47–10.44)f	13.83 (12.12–15.74)<br>8.84 (7.47–10.44)f	x<br>x	n/a<br>n/a	12.63 (11.99–14.48)f<br>8.11 (6.79–9.67) f	12.63 (10.99–14.48)f<br>8.11 (6.79–9.67)f	x<br>x

Country	Boys, 95% CI, %				Girls, 95% CI, %			
	**2000–2009**	**2010–2020**	**2000–2020**	**Trend**	**2000–2009**	**2010–2020**	**2000–2020**	**Trend**
**SVN**	18.27 (17.94–18.61)f<br>5.95 (5.75–6.15)f	21.67 (17.09–27.06)<br>7.27 (6.75–7.83)f	20.39 (16.90–24.38)<br>6.83 (4.80–9.62)	3.4*<br>1.32*	18.13 (17.79–18.47)f<br>6.06 (5.85–6.28)f	18.21 (13.59–23.98)<br>5.50 (2.47–11.80)	18.11 (14.92–21.82)<br>5.78 (3.74–8.82)	0.08*<br>–0.56*
**ESP**	25.78 (21.43–30.66)<br>7.34 (5.30–10.09)	23.72 (22.88–24.58)f<br>7.58 (5.33–10.69)	24.82 (22.38–27.42)<br>7.60 (6.17–9.33)	–2.06*<br>0.24*	24.18 (20.35–28.48)<br>5.81 (4.10–8.17)*	24.53 (23.66–25.41)f<br>7.91 (4.82–12.72)	24.41 (21.99–27.01)<br>6.92 (5.32–8.83)	0.35<br>2.1*
**SWE**	15.96 (13.25–19.10)<br>2.95 (2.60–3.35)f	16.32 (13.66–19.39)f*<br>2.95 (1.89–4.59)f	16.06 (14.01–18.35)*<br>2.95 (2.61–3.33)f	0.36<br>0.01	16.20 (13.69–19.08)<br>3.31 (2.40–4.55)	21.16 (18.27–24.38)f<br>3.21 (2.12–4.83)f	16.95 (14.79–19.35)<br>3.27 (2.38–4.48)	4.96*<br>–0.1
**CHE**	17.02 (13.10–21.83)<br>3.88 (2.53–5.92)	13.51 (12.51–14.57)f<br>3.21 (2.72–3.79)f	15.86 (13.27–18.84)<br>3.66 (2.62–5.09)	–3.51<br>–0.67	16.93 (13.36–21.23)<br>3.99 (2.59–6.09)	14.05 (13.04–15.12)f<br>3.15 (2.67–3.72)f	16.00 (13.39–19.02)<br>3.75 (2.53–5.52)	–2.88<br>–0.84
**TUR**	15.04 (12.55–17.94)<br>2.70 (1.96–3.70)	27.85 (25.45–30.38)f<br>10.85 (9.26–12.68)f	16.80 (14.72–19.11)<br>3.52 (2.70–4.59)*	12.8*<br>8.15*	14.99 (12.70–17.62)<br>3.11 (2.25–4.27)*	16.58 (14.58–18.79)f*<br>9.62 (8.02–11.41)f	15.33 (13.39–17.51)*<br>3.72 (2.74–5.05)	1.59<br>6.51*
UKR	n/a<br>n/a	13.01 (10.46–16.17)<br>3.58 (2.29–5.75)	13.01 (10.51–16.10)<br>3.57 (2.43–5.21)	x<br>x	n/a<br>n/a	12.71 (9.78–16.37)<br>2.85 (2.57–3.15)f	12.70 (10.19–15.73)<br>2.85 (2.57–3.15)f	x<br>x

Abbreviation– (See in the Supporting Information Table S1): f–Fixed effects; (note: Random effects in all other results); *–Statistical significance, p<0.005; n/a–Not applicable, x–No result.

Note: Countries that have data available for both decades are in bold; in each country column top row show overweight and bottom row obesity results.

Table S8. Prevalence by Koppen–Geiger climate zones through 2000–2020 and overall (pool estimate (95% confidence interval) of obesity and overweight in European children aged 6–14 years of included studies

Climate	2000–2009			2010–2020			2000–2020			Trends
	N/Cn	95% CI	I^2	N/Cn	95% CI	I^2	N/Cn	95% CI	I^2	
	**Boys**									
Hot	35/6	21.71 (20.39–23.11)	95.7	22/8	22.91 (21.38–24.51)	97.09	57/9	22.20 (21.29–23.14)	96.31	1.2
		7.62 (6.73–8.61)	96.6		9.11 (7.89–10.43)	97.98		8.23 (7.55–8.96)	96.03	1.49
Warm	20/6	16.70 (15.29–18.32)	94.6	14/8	17.51 (14.29–21.3)	98.8	34/10	16.79 (14.98–18.79)	98.69	0.81
		4.99 (4.11–6.05)	95.8		7.11 (5.11–9.92)	98.91		5.76 (4.87–6.79)	98.04	2.12
Temperate	28/13	13.98 (12.10–16.09)	96.7	15/11	13.99 (12.62–15.51)	91.09	43/16	14.03 (12.69–15.42)	96	0.01
		3.72 (2.91–4.74)	95.8		4.90 (3.88–6.13)	93.96		4.11 (3.47–4.87)	95.25	1.18
Cold	5/3	13.30 (10.61–16.39)	72	4/2	15.11 (12.59–17.91)	63.83	9/4	14.19 (12.54–16.11)	65.24	1.81
		3.08 (2.48–3.82) f	34.4		4.61 (2.40–8.72)	88.39		3.58 (2.39–5.33)	84.33	1.53
		**Girls**								
Hot	35/6	21.32 (19.88–22.71)	96.1	22/8	21.21 (19.30–23.33)	98.47	57/9	21.31 (20.19–22.43)	97.52	–0.11
		6.52 (5.61–7.43)	96.9		7.89 (6.49–9.63)	98.73		7.02 (6.30–7.82)	97.96	1.37
Warm	20/6	17.41 (15.79–19.22)	95.7	14/8	16.91 (14.89–19.03)	96.51	34/10	17.09 (15.7–18.61)	97.78	–0.5
		5.39 (4.59–6.42)	94.1		5.59 (4.58–6.79)	96.01		5.58 (4.99–6.24)	95.14	0.2
Temperate	28/13	13.50 (11.71–15.63)	96.6	15/11	14.49 (13.21–15.92)	89.25	43/16	13.92 (12.62–15.23)	95.44	0.99
		3.29 (2.59–4.02)	92.4		4.41 (3.61–5.43)	92.22		3.66 (3.15–4.25)	92.31	1.12
Cold	5/3	12.09 (9.98–14.51)	57.2	4/2	17.40 (15.89–19.02) f	49.12	9/4	14.39 (12.22–16.89)	79.39	5.31
		2.11 (1.22–3.81)	67		4.91 (2.80–8.39)	86.69		3.28 (2.17–4.92)	82.59	2.8

No statistical significance between genders and trends

Note: In each climate column top row show overweight and bottom row obesity results.

Figure S1. European map of the number of studies per country and region

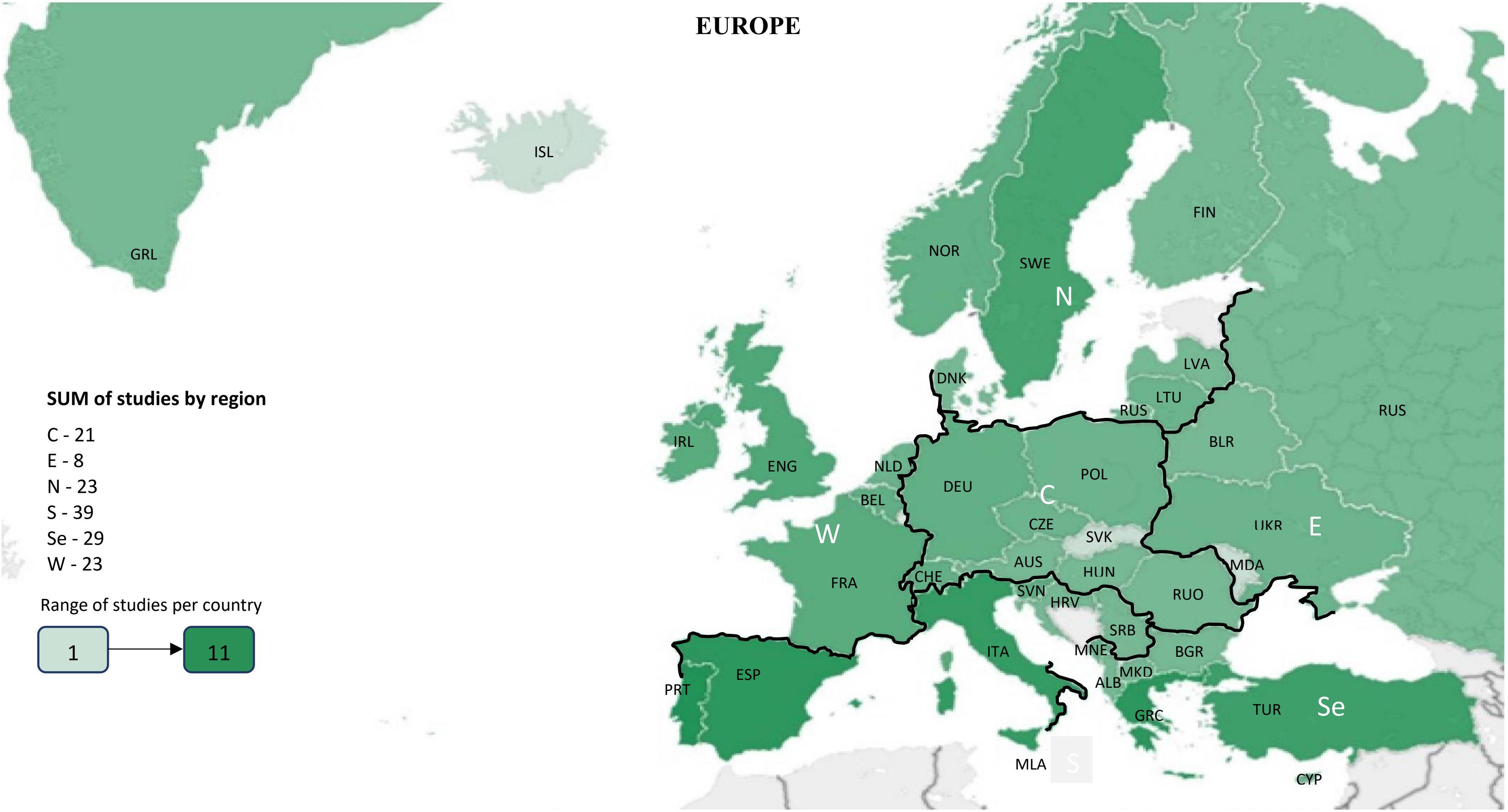

Description: European map are shaded by number of studies per country in green color.
Abbrevation: C-Central; E-East; N-North; S-South; Se-Southeast; W-West.
Note: The three-letter country abbreviations can be found in Table S1.

Figure S2. **Pooled estimate for the prevalence from 2000 to 2020 by gender of (A) overweight and (B) obesity in children aged 6–14 years across 18 European countries according to IOTF defnition criteria**

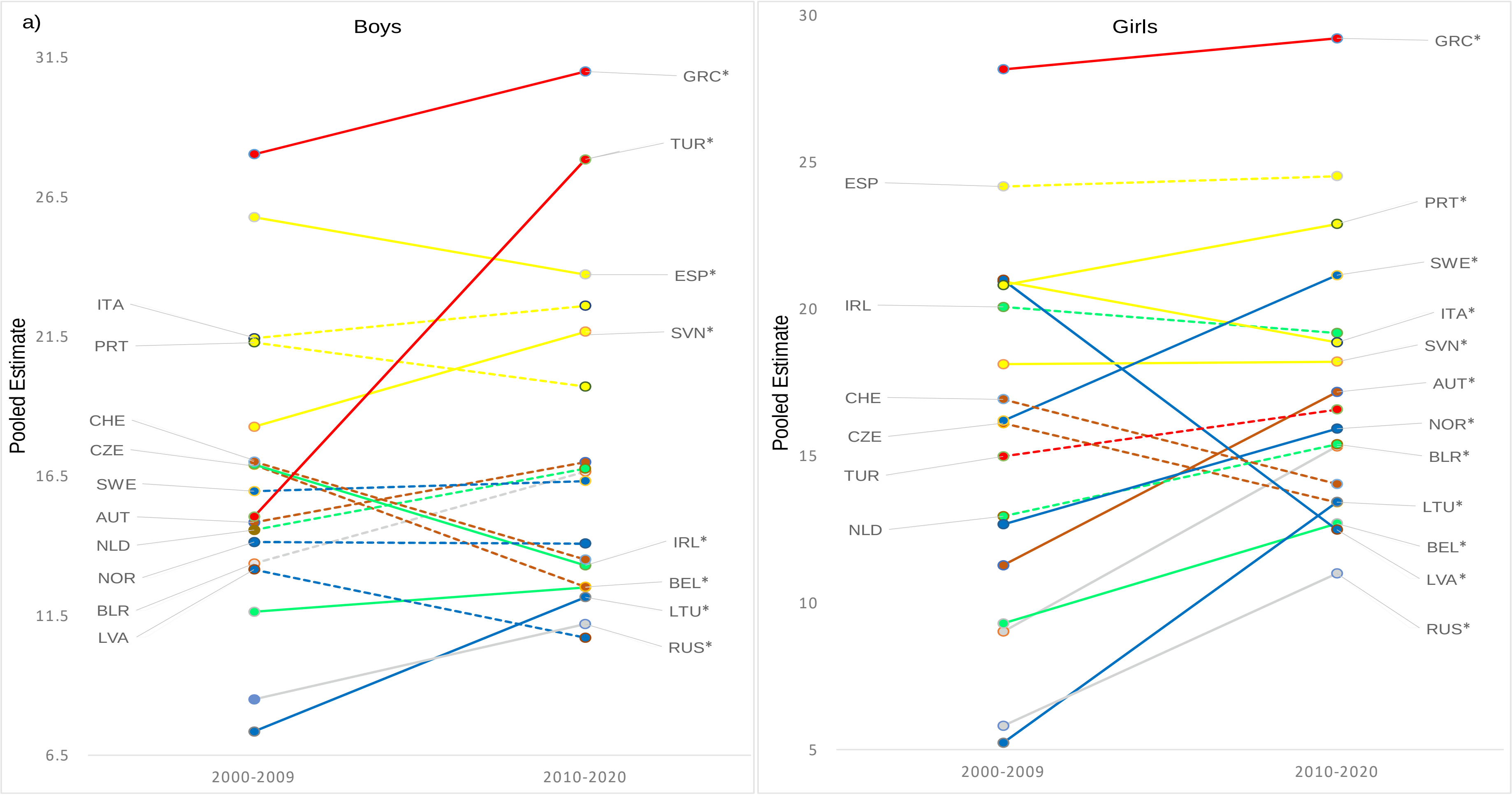

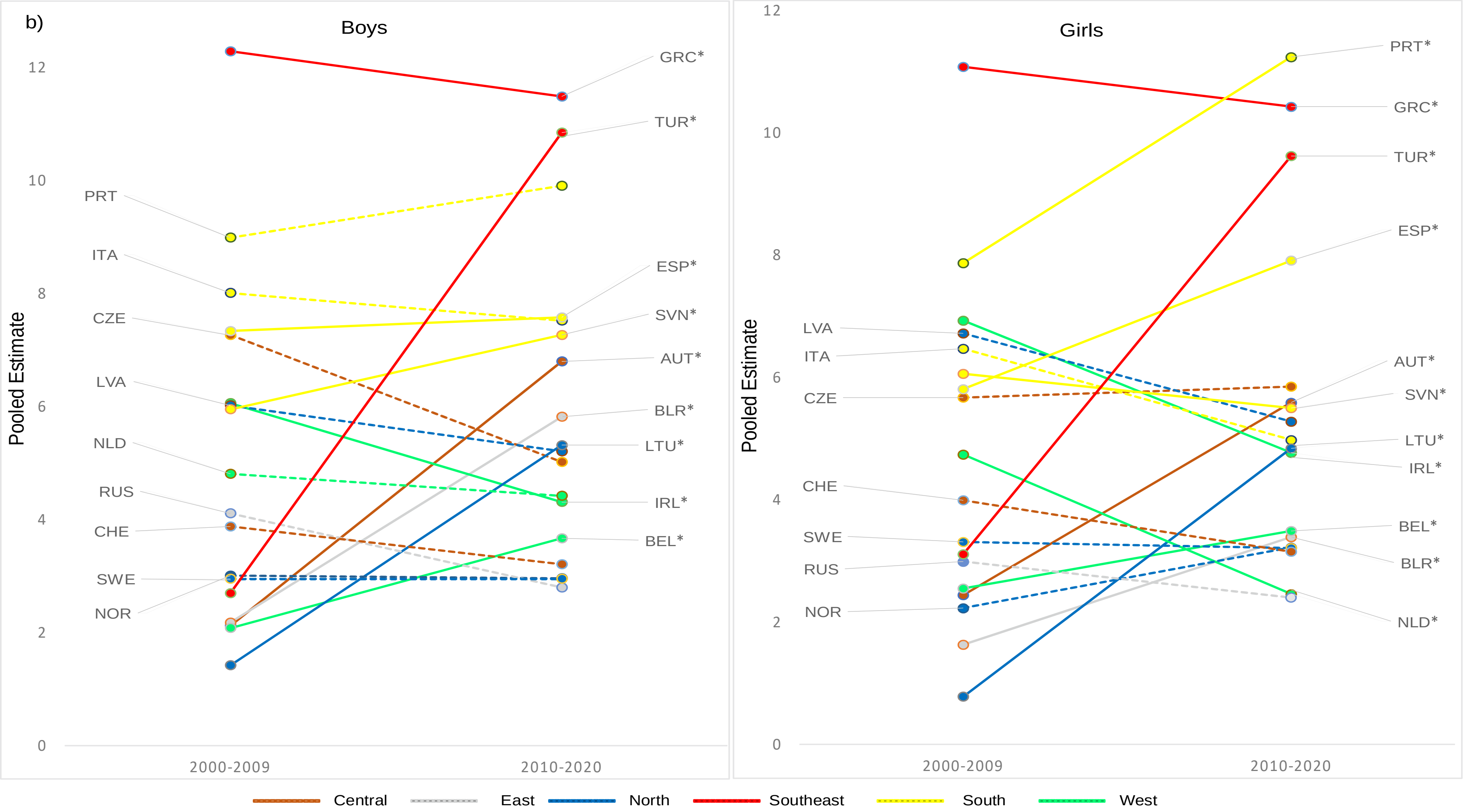

*Note: On the right side are countries for which a full line and * show statistical sagnificance, while on the left side lines with dots show a lack of significant results in the prevalence of differences between two decades; The three-letter country abbreviations on the right and left side can be found in Table S1; All countries belonging to a particular region are marked with the color of that region.*

Figure S3. **Pooled estimate for the prevalence accros Koppen–Geiger climate zones by gender of (A) overweight and (B) obesity in children aged 6–14 years across European countries according to IOTF defnition criteria**

a)

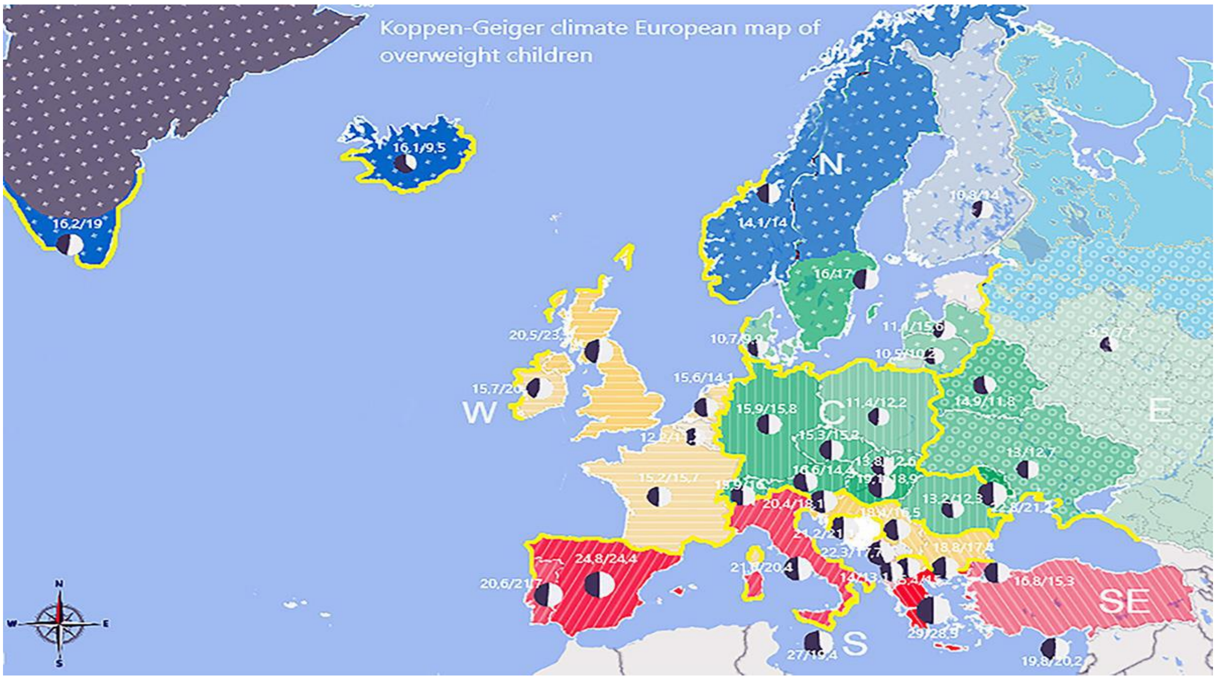

b)

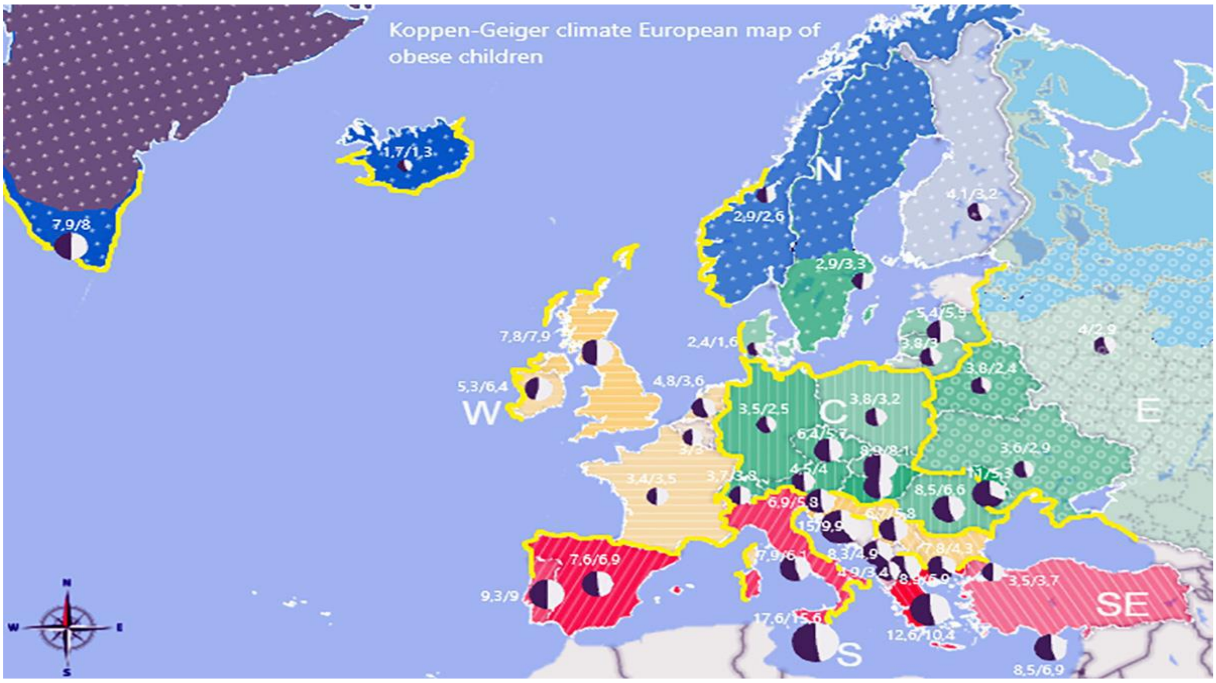

Description: Pooled estimate prevalence ratio between genders – circles in European countries with black parts for boys and white parts for girls. The borders of 6 European regions are marked with a full yellow line. State of prevalence of obesity and overweight are shown through diferent shades of 4 colors for Koppen–Geiger climate zones. Climate zone colors are: Red–Hot; Orange–Warm; Green–Temperate; and Blue–Cold; Additional Region markings: Full stars–North; Hollow circles–East; Vertical lines–Central; Horizontal lines–West; Left slope lines–South; and Right slope lines–Southeast.

Figure S4. Pooled estimate for the prevalence from 2000 to 2020 by gender of (A) overweight and (B) obesity in children aged 6–14 years across 4 Koppen–Geiger climate zones according to IOTF defnition criteria

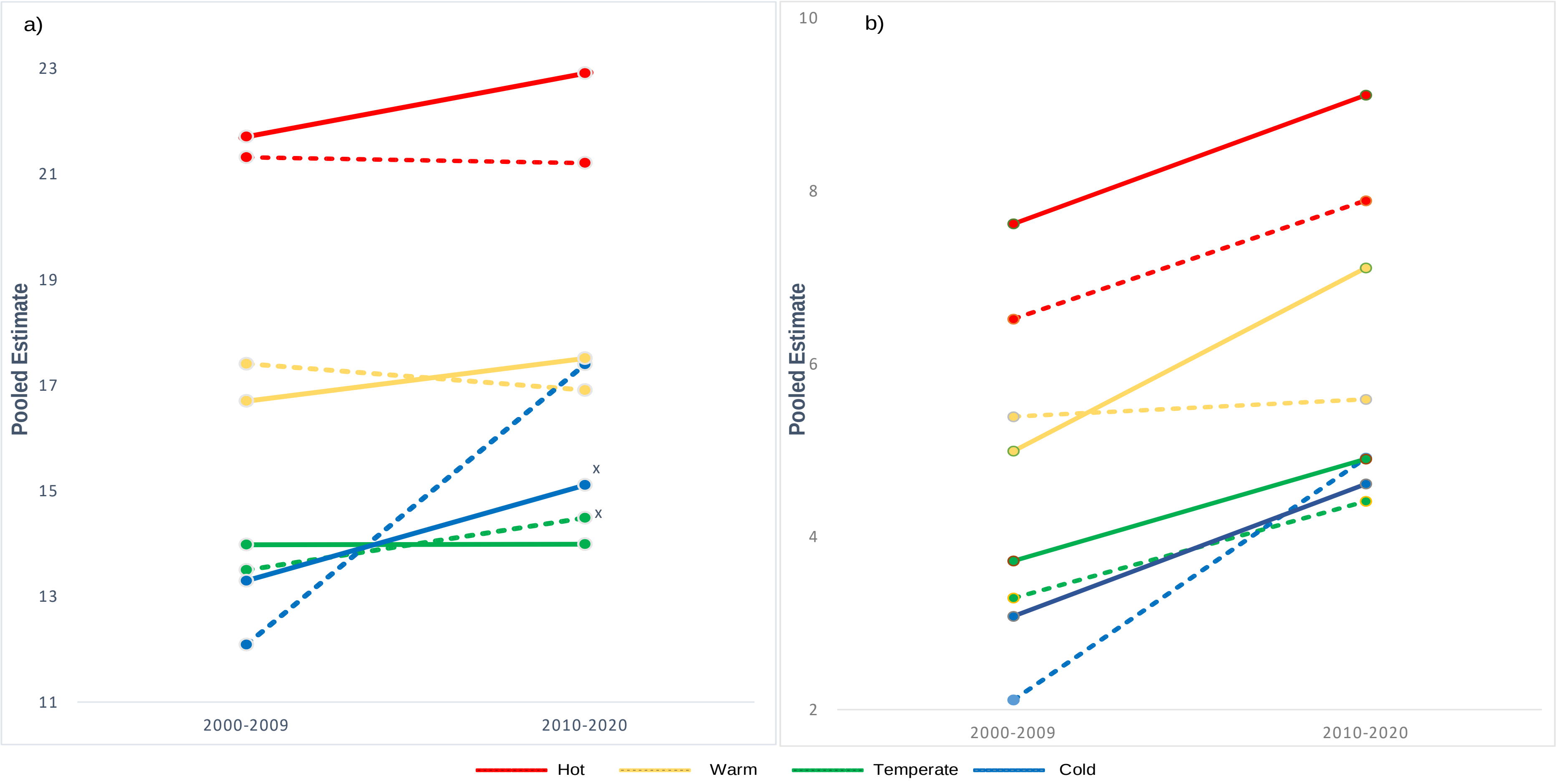

Note: Full line represent boys and line with dots represent girls, statistically no significant results of prevalence differences between two decades are marked with an x on the right side.
